# Translational repression by a miniature inverted-repeat transposable element in the 3′ untranslated region

**DOI:** 10.1038/ncomms14651

**Published:** 2017-03-03

**Authors:** Jianqiang Shen, Juhong Liu, Kabin Xie, Feng Xing, Fang Xiong, Jinghua Xiao, Xianghua Li, Lizhong Xiong

**Affiliations:** 1 National Key Laboratory of Crop Genetic Improvement and National Center of Plant Gene Research (Wuhan), Huazhong Agricultural University, Wuhan 430070, China; 2 College of Plant Science and Technology, Huazhong Agricultural University, Wuhan 430070, China

## Abstract

Transposable elements constitute a substantial portion of eukaryotic genomes and contribute to genomic variation, function, and evolution. Miniature inverted-repeat transposable elements (MITEs), as DNA transposons, are widely distributed in plant and animal genomes. Previous studies have suggested that retrotransposons act as translational regulators; however, it remains unknown how host mRNAs are influenced by DNA transposons. Here we report a translational repression mechanism mediated by a stowaway-like MITE (sMITE) embedded in the 3′-untranslated region (3′-UTR) of *Ghd2,* a member of the CCT (CONSTANS [CO], CO-LIKE and TIMING OF CAB1) gene family in rice. *Ghd2* regulates important agronomic traits, including grain number, plant height and heading date. Interestingly, the translational repression of Ghd2 by the sMITE mainly relies on Dicer-like 3a (OsDCL3a). Furthermore, other MITEs in the 3′-UTRs of different rice genes exhibit a similar effect on translational repression, thus suggesting that MITEs may exert a general regulatory function at the translational level.

## Abstract (web summary)

Miniature inverted-repeat transposable elements (MITEs) are common components of eukaryotic genomes. Here the authors show that a stowaway-like MITE in the 3′-untranslated region of the rice *Ghd2* gene can suppress translation in a DCL3a-dependent manner representing a novel role for transposons in translation.

Transposable elements (TEs), as parasitic DNA, have the ability to spread themselves throughout genomes, causing disruption of gene function and driving genome evolution^1^,^2^. In eukaryotic genomes, TEs are classified as retrotransposons (Class I) intermediated by RNAs, and DNA transposons (Class II) that move directly in a ‘cut and paste' mode^3^. Among these transposons, nonautonomous TEs propagate via their autonomous partners that allow their accumulation in animal and plant genomes^4^,^5^,^6^,^7^,^8^.

With the advances of genomic and transcriptomic sequencing, TEs, especially retrotransposons^9^, have been identified as gene regulators. In mammalian TE studies, short-interspersed elements (SINEs) or long-interspersed elements (LINEs) were demonstrated to modulate gene transcription through restructuring chromatin epigenetically^10^,^11^ and function as promoters or enhancers^12^,^13^. In addition, the retrotransposons, transcribed as part of mRNAs, could influence the alternative splicing of transcripts^14^,^15^, alternative polyadenylation^16^,^17^, localization^18^, stability^19^, as well as translation^20^,^21^,^22^,^23^. For example, a subset of human transcripts contain inverted repeat Alu elements (IRAlus) in the 3′-UTR, and these elements recruit dsRNA-dependent protein kinase (PKR) to translation initiation factor 2α for subsequent phosphorylation, which ultimately leads to translational repression^20^,^24^. Meanwhile, the responses of mammalian SINEs to stresses cause translational enhancement^25^. In addition, the function of SINEUPs as long noncoding RNAs in stimulating protein synthesis depends on SINEB2 repeats which are complementary to the 5′-ends of target mRNAs^26^.

As a class of DNA transposons, MITEs constitute ∼5.2% of the rice genome sequence, which are distributed non-randomly throughout the genome and enriched in gene regions in particular^27^. Some MITEs within or near gene-coding regions play regulatory roles in modulating mRNA transcription. Previous studies reported that the DNA transposon mPing tends to insert into the 5′ flanking sequences of genes, causing inducible expression of adjacent genes in response to stresses^28^. In addition, the methylation of MITE-embedded promoters has the potential to silence the transcription of nearby genes. Recently, rice 24-nucleotide (24-nt) small interfering RNA (siRNAs), dependent on OsDCL3a, have been identified as potential silencers for the expression of multiple genes that bear MITEs in their promoter regions^29^. Meanwhile, a previous study reported that a MITE in the promoter would repress the gene expression via RNA-directed DNA methylation and H3K9 dimethylation probably^30^. Other studies also support that MITEs could regulate gene transcription on a genome-wide scale^31^,^32^. However, it remains unknown whether MITE-contained transcripts could regulate the host gene expression at the translational level in plants.

Here we demonstrate that a stowaway-like MITE represses the protein synthesis of an agronomically important gene *Ghd2*, which controls grain number, plant height and heading date in rice (*Oryza sativa*). The translational repression by the MITE depends on Dicer-like 3a. In addition, the MITE embedded in 3′-UTR of other genes has the similar function in the translational repression. Our findings reveal an unreported role of DNA transposons in the regulation of gene expression at the translational level.

## Results

### Ghd2 functions as a suppressor in flowering time in rice

Previous studies have demonstrated that CCT domain-containing genes regulate diverse biological processes, including developmental controls and environmental responses in plants^33^,^34^,^35^,^36^. Natural variations in CCT domain-containing genes are also associated with agronomic traits and geographic distribution in cereals^36^,^37^,^38^. In an effort to identify agronomically important genes in the CONSTANS-like gene family in rice, we found that a member (LOC_Os02g49880) of this gene family had functions (as the description below) similar to its close homolog *Ghd7* (ref. 36), which controls grain number, plant height and heading date. This gene was once simply named *OsK* in a phylogenetic study^39^. Considering the standard gene nomenclature on the basis of gene-controlled phenotypes in rice^40^, it was renamed *Ghd2*.

**Figure 1:**
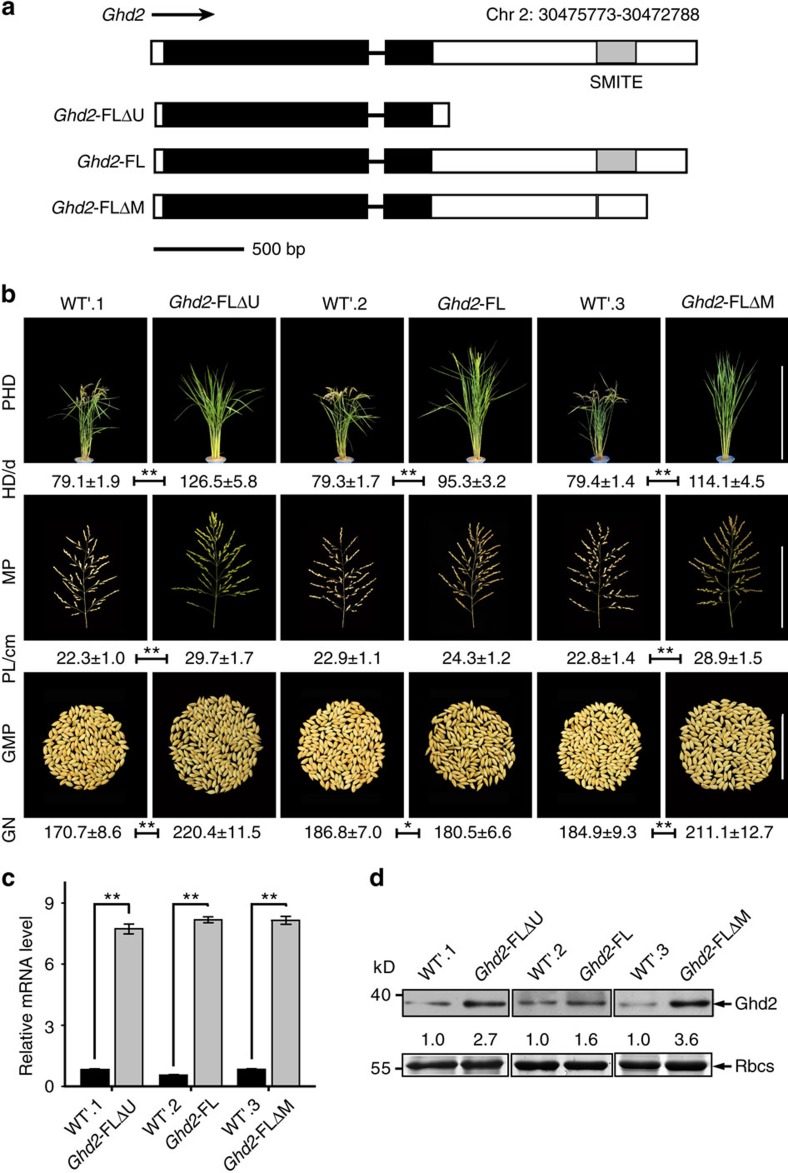
The *Ghd2* 3′-UTR acts as a negative regulator of flowering and yield. (**a**) *Ghd2* genomic organization. Exons are in black; 3′- and 5′-UTRs are in white; the transposable element (sMITE) is in grey; and the intron is indicated as a line. (**b**) Phenotypes of *Ghd2*-FLΔU overexpression and wild-type (WT'1) lines, *Ghd2*-FL overexpression and wild-type (WT'2) lines, and *Ghd2*-FLΔM overexpression and wild-type (WT'3) lines. GN, grain number of main panicle; GMP, grains of main panicle; HD, heading date (days); MP, main panicle; MPL, main panicle length (cm); PHD, phenotype of heading date at the maturation stage of WT. Scale bar: 1 m (PHD), 20 cm (MP), 10 cm (GMP). WT' indicates segregated non-transgenic lines. The data represent the means±s.d., *n*=20. *P<0.05, **P<0.01 (Student's *t*-test). (**c**) Quantitative expression of *Ghd2* mRNA in *Ghd2*-FLΔU and WT'1; *Ghd2*-FL and WT'2; and *Ghd2*-FLΔM and WT'3 lines. The data represent the means±s.d., *n*=3. **P<0.01 (Student's *t*-test). (**d**) Ghd2 protein levels in *Ghd2*-FLΔU and WT'1; *Ghd2*-FL and WT'2; and *Ghd2*-FLΔM and WT'3 lines were measured by western blotting with an anti-Ghd2 antibody. Rubisco (Rbcs) was loaded as a control. The numbers between two blots indicate relative abundance of Ghd2 normalized by Rbcs for single samples.

In contrast to other members in the CCT gene family, *Ghd2* contains a MITE in its 3′-UTR. Therefore, *Ghd2* may be an excellent candidate to investigate the possible roles of MITEs in the gene regulation. This MITE, which is a 217-nt inverted region (IR), was identified as a copy of a stowaway-like MITE designated as stowaway38 (OsT38)^41^. By using rapid amplification of cDNA ends (RACE), the transcriptional termination site (TTS) of the *Ghd2* mRNA was mapped to 340 nucleotides downstream of the sMITE location (Fig. 1a). To determine whether the sMITE in the *Ghd2* 3′-UTR has functional significance, we overexpressed the full-length *Ghd2*, including its 3′-UTR (*Ghd2* FL), and the complete coding sequence without the 3′-UTR (*Ghd2* FLΔU) in the Zhonghua11 rice variety (Fig. 1a). Furthermore, we examined the phenotypes of single-copy transgenic lines. Interestingly, the *Ghd2* FLΔU overexpression lines showed approximately 60-day delay in flowering time, whereas the *Ghd2* FL overexpression lines exhibited nearly 15-day delay (Fig. 1b and Supplementary Table 1). In addition, the *Ghd2* knockout lines with reading-frame-shifted mutations generated by the CRISPR/Cas9 technique flowered approximately 7-days earlier than the wild-type (WT) lines (Supplementary Fig. 1b).

A quantitative reverse transcription–PCR (qRT–PCR) analysis demonstrated that the *Ghd2* gene was highly expressed in leaves at the reproductive stage (Supplementary Fig. 2) and the expression peaked at 20:00 during a diurnal cycle (Supplementary Fig. 3a and b). Furthermore, the expression level of the *Early heading date 1* (*Ehd1*) gene^42^, but not *Heading date 1* (*Hd1*)^43^, was dramatically reduced in the *Ghd2* FLΔU overexpression lines, which suggested that *Ghd2* may function as a suppressor of *Ehd1*, thus delaying flowering time in rice (Supplementary Fig. 3e and f). In addition to the delayed flowering time, *Ghd2* overexpression caused a significant increase in panicle size, the number of spikelets and grain yield (Fig. 1b), which is similar to the phenotypes of the *Ghd7* overexpression transgenic rice.

### The sMITE functions as a translational repressor

The phenotypic variance between *Ghd2* FL and *Ghd2* FLΔU prompted us to propose that the *Ghd2* 3′-UTR may function as a repressor. To test this hypothesis, we quantified the Ghd2 transcript and protein levels in the *Ghd2* FL and *Ghd2* FLΔU overexpression lines. Interestingly, equivalent levels of *Ghd2* mRNA were observed in the *Ghd2* FL and *Ghd2* FLΔU overexpression lines (Fig. 1c), but the Ghd2 protein level was higher in the *Ghd2* FLΔU overexpression line (3-fold increase compared to the WT) than that in the *Ghd2* FL overexpression line (1.6-fold increase compared to the WT; Fig. 1d). These results imply that the 3′-UTR may regulate *Ghd2* at the translational level.

**Figure 2:**
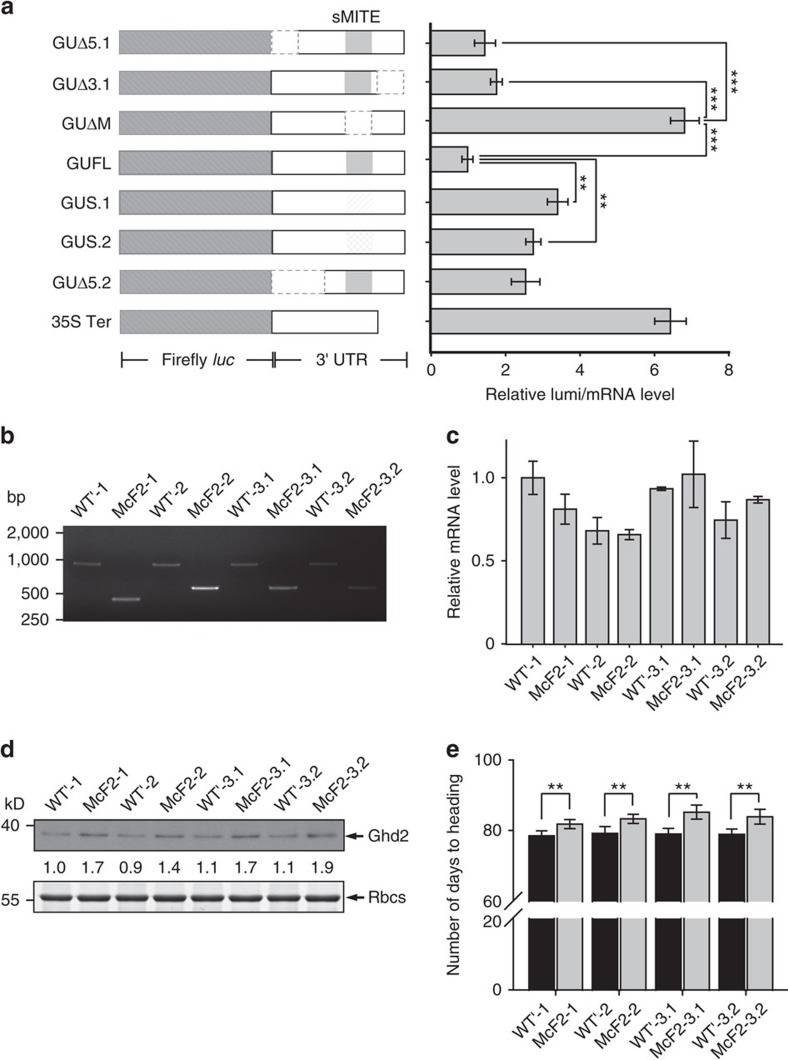
The *Ghd2* 3′-UTR regulates the Ghd2 protein level via a sMITE. (**a**) Left panel, firefly luciferase reporter constructs fusing the full-length *Ghd2* 3′-UTR (GUFL), deletion variants (GUΔ5.1, GUΔ3.1, GUΔM and GUΔ5.2), substitute variants (GUS.1 and GUS.2), and the cauliflower mosaic virus 35S terminator (35S Ter, control). The luciferase genes are in dark grey with diagonals; the 3′-UTRs are in white, the deleted regions are shown with dotted lines, the variants are in light grey with diagonals; and the grey rectangle in the 3′-UTR indicates the sMITE. Right panel, relative luminescence units versus the transcript levels quantified by qPCR (GUFL was normalized to 1). The data in the right panel represent the means±s.d., *n*=3. **P<0.01 (Student's *t*-test). The data indicate the relative luminescence/mRNA level calculated from three independent experiments. (**b**) Genotypes of the multi-sgRNA-mediated transposable element excision lines. Three independent sMITE-excision lines containing 519, 397 and 397 bp deletion, respectively in the *Ghd2* 3′ UTR were backcrossed with wild type to generate the segregated excision (McF2-1, McF2-2, and McF2-3) and non-excision (WT') lines. McF2-3.1 and McF2-3.2 are two backcrossed excision lines from the same transgenic events. (**c**) Quantitative expression of *Ghd2* mRNA in the sMITE excision lines and negative controls. The data represent the means±s.d., *n*=3. (**d**) The Ghd2 protein level in the sMITE excision lines and negative controls was measured by western blotting with the anti-Ghd2 antibody. Rubisco (Rbcs) was loaded as a control. The numbers between two blots indicate relative abundance normalized by Rbcs for single samples. (**e**) The heading date of MITE excision (McF2) and the segregated wild-type (WT') lines. The data represent the means±s.d., *n*=10. **P<0.01 (Student's *t*-test).

To investigate whether the sMITE regulates Ghd2 translation directly, we transiently expressed the *Ghd2* 3′-UTR in rice protoplasts using a dual luciferase reporter assay. Constructs of a firefly luciferase gene bearing the *Ghd2* 3′-UTR deletion forms or the cauliflower mosaic virus 35S terminator (35S Ter) were used as reporters. A *Renilla* luciferase gene with 35S Ter in its 3′-UTR was co-transfected to normalize the transfection efficiency (Fig. 2a). The relative luminescence normalized to the mRNA level of the luciferase gene (relative luminescence/mRNA level) was used to evaluate the relative translational efficiency. The results showed that the complete *Ghd2* 3′-UTR and the shortened 3′-UTRs containing the sMITE significantly repressed luciferase expression, but the partial 3′ UTRs lacking the sMITE had no repression effect (Fig. 2a). Furthermore, the constructs with sMITE substituted by an unrelated sequence (GUS.1) or another retrotransposon (GUS.2) were assayed. Compared to the construct with the complete 3′ UTR, the constructs with GUS.1 or GUS.2 exhibited an approximate 3-fold increase in the relative luminescence/mRNA level (Fig. 2a). These results indicate that the sMITE is a functional TE required for the 3′-UTR-mediated translational repression of Ghd2. To further confirm the results of the transient expression, we overexpressed the full-length *Ghd2* gene without the sMITE (*Ghd2* FLΔM) in rice. As expected, the single-copy transgenic lines of *Ghd2* FLΔM and *Ghd2* FLΔU exhibited similar phenotypes (Fig. 1b), *Ghd2* mRNA levels (Fig. 1c), and Ghd2 protein abundance (Fig. 1d).

To further clarify the regulatory role of sMITE in Ghd2 translation, we generated transgenic rice lines, in which the sMITE genomic sequence was precisely excised from the *Ghd2* 3′-UTR using CRISPR/Cas9 editing (Fig. 2b and Supplementary Fig. 4a). To segregate the foreign DNA from the sMITE excision plants, three T_2_ excision lines with two different sizes of excision fragment (Supplementary Fig. 4b) were backcrossed with the wild type. The segregated sMITE excision plants (named McF2-1, McF2-2 and McF2-3) and the wild type (WT') from BC_1_F_2_ generation were identified for Ghd2 expression level analysis. The *Ghd2* mRNA levels in the sMITE-excised lines showed no significant change compared to that in the non-excision lines (Fig. 2c and Supplementary Fig. 4c), while the Ghd2 protein levels in the sMITE-excised lines were obviously higher (Fig. 2d and Supplementary Fig. 4d). Meanwhile, the flowering time of the excision lines showed approximately 5-day delay compared to that of the wild type (Fig. 2e). These results further confirmed that *Ghd2* translation is repressed by the sMITE in its 3′-UTR.

### Translational repression by the sMITE depends on OsDCL3a

It has been reported that transposable element-derived siRNAs mediate chromatin modification to regulate the abundance of *FLC* mRNA^44^,^45^ and alter ribosome sensitivity to repress translation^46^. We identified two siRNA clusters, siR381 and siR382, derived from the sMITE using the Cereal Small RNA database^47^. To investigate whether these two siRNAs are involved in the *Ghd2* translational repression, we overexpressed them in rice. Surprisingly, neither the *Ghd2* mRNA nor the Ghd2 protein level showed significant differences between the siRNA overexpression lines and the WT lines (Supplementary Fig. 5). These results suggest that the translational repression may not be related to the abundance of the siRNAs derived from the sMITE.

**Figure 3:**
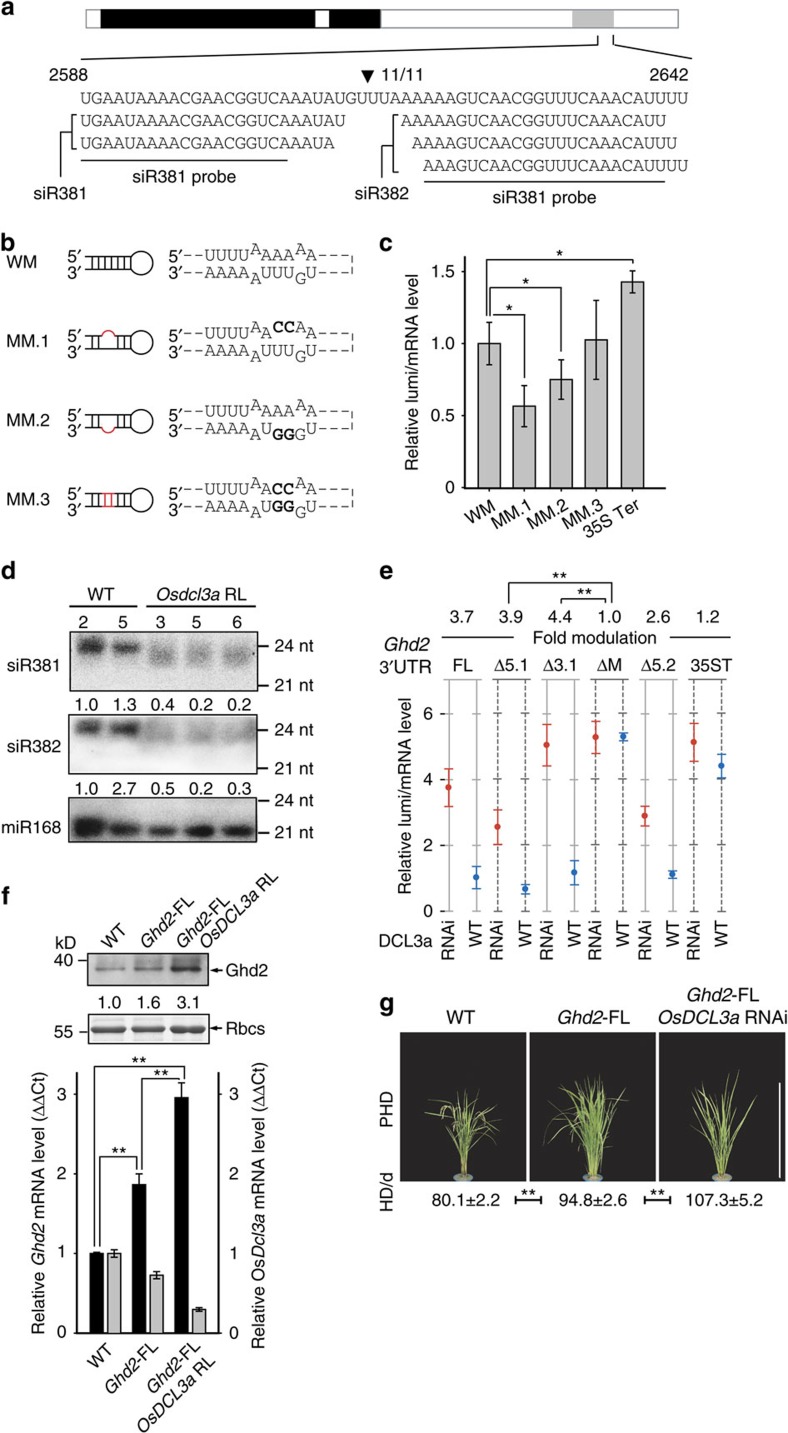
SMITE-mediated *Ghd2* translational repression depends on OsDCL3a. (**a**) The cleavage site in the sMITE (indicated by a black triangle) was mapped by RLM-5′ RACE. The sequence indicates the 2588 to 2643nt of the *Ghd2*. The small RNA sequences below were aligned to the sMITE in the *Ghd2* 3′-UTR. Probes used in the small RNA northern blotting are underlined. (**b**) Predicted secondary structure of the wild-type (MW) and mutant (MM.1-MM.3) sites. The right panel shows the site mutants (bold). (**c**) Relative luminescence units versus the transcript levels normalized to MW (set as 1). The data represent the means±s.d., *n*=3. *P<0.05 (Student's *t*-test). The data indicate the relative abundance calculated from three independent experiments. (**d**) Decreased levels of siRNAs from the sMITE in the Osdcl3a RNAi lines (RL). The probes for the siRNAs are as shown in **a**. MiR168 was used as a loading control. The numbers below the blots indicate the relative abundance. (**e**) Dual luciferase reporter assay of the *Ghd2* 3′ UTR and its deletion variants in the Osdcl3a RNAi lines and the WT. Relative luminescence units versus their relative mRNA level are shown (normalized to GUFL). Fold modulation means the relative luminescence/mRNA level of *OsDCL3a* RNAi line versus the WT. The data represent the means±s.d., *n*=3. **P<0.01 (Student's *t*-test). (**f**) The *Ghd2* mRNA and protein levels were quantified in the WT, *Ghd2* FL and *OsDCL3a*-RNAi *Ghd2* FL lines. The Ghd2 protein was measured by western blotting using the anti-Ghd2 antibody. Rubisco (Rbcs) was loaded as a control. The numbers between two blots indicate relative abundance for single samples. The expression of *Ghd2* mRNA was quantified by quantitative RT–PCR. The data represent the means±s.d., *n*=3. **P<0.01 (Student's *t*-test). (**g**) Phenotypes of the WT, *Ghd2* FL and *OsDCL3a*-RNAi *Ghd2* FL lines. HD, heading date (days); PHD, phenotype of the heading date at the maturation stage of the WT (scale bar, 50 cm). The data represent the means±s.d., *n*=10. **P<0.01 (Student's *t*-test).

We further examined whether the translational repression of Ghd2 was associated with the siRNA biogenesis. A modified RNA ligase-mediated rapid amplification of 5′ cDNA ends (RLM-RACE) was employed to examine the target site in *Ghd2* 3′-UTR transcripts *in vivo*. The result revealed the presence of a cleavage site, which is located between the two siRNA sequences (Fig. 3a). To assess the importance of this site for translation, the sMITE and its mutated forms of the site (Fig. 3b) were fused to the 3′-UTR of firefly luciferase gene for expression assay. Compared to the control (35S Ter), the luciferase translation was repressed in the protoplasts transfected with the wild-type sMITE construct (WM; Fig. 3c). The translational repression in the protoplasts transfected with the mutant constructs (MM.1 and MM.2) was enhanced compared to that with the WM (Fig. 3c). This result may be due to the mutations that are possibly more effectively recognized and cleaved by endoribonucleases, such as dicer-like proteins (DCL)^48^.

The increased suppression of the mutated luciferase reporters prompted us to investigate whether endoribonucleases, which process the siRNAs, are responsible for the translational repression. Small RNA northern blot analyses were preformed in RNA interference (RNAi) or mutant lines of Dicer-like endoribonuclease genes including *OsDCL1*, *OsDCL3a*, *OsDCL3b* and *OsDCL4*. Only in the OsDCL3a RNAi lines, the abundance of the sMITE-derived 24 nt-siRNAs was attenuated (Fig. 3d and Supplementary Fig. 6). The results revealed that OsDCL3a may act as a regulator for the sMITE-derived siRNAs. Heterochromatic siRNAs are derived from double-stranded RNA (dsRNA) generated by RDR2, processed by DCL3 and incorporated into AGO4 in plants^49^,^50^,^51^,^52^. Therefore, we speculated that some of the proteins involved in siRNA biogenesis and binding, such as OsRDR2, OsDCL3a, OsAGO4a and OsAGO4b might be involved in sMITE-mediated translational regulation^31^. This hypothesis was tested by examining the *Ghd2* transcript and protein abundance in the mutant or RNAi lines of these genes. Interestingly, compared with the WT line, the *OsDCL1*, *OsDCL3a* and *OsDCL3b* RNAi lines exhibited an increased accumulation of the Ghd2 protein, but not the *Ghd2* transcripts (Supplementary Fig. 7a,b, and c); however, Ghd2 protein was decreased in the *dcl4* mutant (Supplementary Fig. 7d). The knockdown of *OsRDR2* and *OsAGO4ab* had no effect on Ghd2 protein accumulation (Supplementary Fig. 8). All these results indicate that OsDCL3a may be involved in the sMITE-mediated translational repression.

To further confirm this hypothesis, we assayed the relative luminescence/mRNA level of the reporter gene containing the sMITE in the 3′-UTR in the Osdcl3a-RNAi line and WT protoplasts. The relative luminescence/mRNA level of the reporter gene was significantly higher in the Osdcl3a-RNAi lines than in the WT (Fig. 3e). The results indicate that translational repression was weakened in the OsDCL3a RNAi protoplast. Furthermore, the knockdown of *OsDCL3a* in the *Ghd2* FL transgenic plants resulted in phenotypes similar to that of *Ghd2* FLΔM (Fig. 3g). The Ghd2 protein level was higher in the *OsDCL3a*-RNAi *Ghd2* FL plants than in the *Ghd2* FL plants (Fig. 3f). Interestingly, the *Ghd2* transcript level was increased in the *OsDCL3a* RNAi line, suggesting that the *Ghd2* mRNA may be more stable because of *OsDCL3a* deficiency (Fig. 3f). These results together suggest that OsDCL3a is responsible for Ghd2 translational repression mediated by the sMITE.

### MITEs in other 3′-UTRs also repress translation

**Figure 4:**
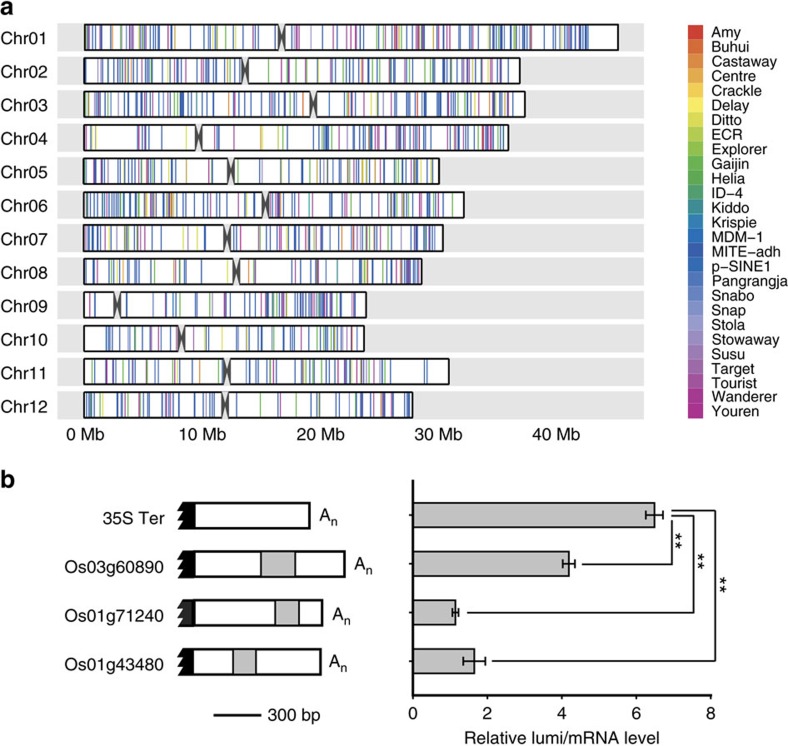
Other MITEs in 3′-UTRs repress translation. (**a**) Genomic distribution of 3′ UTR-localized MITEs in the rice genome. A karyogram composed of colored bars is presented; each colored bar represents a MITE in the 3′ UTR of the target gene. Black angles represent centromeres. (**b**) Genomic organization of three randomly chosen MITEs in 3′-UTRs (left) and their effects on translational repression in rice protoplasts (right). The data represent the means±s.d., *n*=3. **P<0.01 (Student's *t*-test). The data indicate relative luminescence/mRNA level based on three independent experiments.

MITEs associated with genes are broadly distributed in the rice genome^41^. For example, the sMITE in this study (OsT38) has ∼4,518 copies (Supplementary Fig. 9). We further investigated whether other MITE-embedded 3′-UTRs have similar roles in the repression of protein synthesis. To find more genes with an insertion of MITE in their 3′-UTR, MITEs identified in the rice genome^53^ were scanned for protein-encoding genes containing a MITE in their 3′-UTRs. A total of 1,182 genes were identified with 3′-UTRs embedded with 1,694 MITEs potentially generating 23- to 24-nt siRNAs (Fig. 4a). Three 3′-UTRs with MITEs other than OsT38 were randomly chosen, fused to the 3′-UTR of the luciferase reporter, and transfected into rice protoplasts. All three 3′-UTRs prominently repressed translation when compared with a control reporter carrying the 35S terminator (Fig. 4b). These results imply that a large number of plant genes may be regulated by MITEs at the translational level.

## Discussion

In eukaryotic genomes, retrotransposons have been found to have multiple functions in regulating genes expression and are known to participate in various metabolic processes^9^. In animal and plant genomes, MITEs (a type of nonautonomous DNA transposons) constitute 0.32–15.8% of the genomic sequences^54^. These MITEs are found in untranslated regions of mRNA including 5′-UTRs, introns and 3′-UTRs^27^. Extensive evidence supported that the MITEs present upstream of adjacent genes can alter their transcription as promoters^28^ or through epigenetic modifications^29^. However, little is known about the functions of MITEs in the translational modulation despite the predominant regulatory roles of MITEs at the transcriptional level. In this study, we found that a stowaway-like MITE functions in the translational repression of the *Ghd2* gene, which controls grain number, plant height, and heading date. Furthermore, this translational repression is dependent on OsDCL3a. Based on our results, we propose a simple model for the role of this MITE in the translational repression (Supplementary Fig. 10). Besides *Ghd2*, a genome-wide search identified that 1,182 genes harbour MITEs in their 3′-UTRs, and these genes also appear to be modulated also through MITE-mediated translational repression.

In this study, we found that the MITE-mediated translational repression is dependent on OsDCL3a. It remains unknown which phase of mRNA translation is blocked and how the translation is repressed. Most probably, OsDCL3a may direct the translational repression by processing the MITE nascent transcripts, thus generating a shortened mRNA. The shortened mRNA or the loss of a polyadenylation tail may be a trigger to the translational repression. Several lines of evidence support this hypothesis. First, the cleavage in the MITE suggests that the *Ghd2* mRNA is truncated by OsDCL3a, which was confirmed by the result of dual-luciferase reporter assay with the constructs mutated at the cleavage site (Fig. 3a,b). Second, the *Ghd2* mRNA levels in the OsDCL3a-RNAi *Ghd2* FL lines were higher than that in the Ghd2 FL overexpression lines (Fig. 3f). This indicates that the mRNA truncation can be attenuated due to the suppression of OsDCL3a. Previous study reported that the 24-nt siRNAs derived from transposon transcripts processed by OsDCL3a mediate DNA methylation and result in histone modification (H3K9 dimethylation) in rice^29^. To check whether the DNA methylation and/or H3K9 dimethylation are affected by the MITE at the *Ghd2* locus, bisulfite sequencing and chromatin immunoprecipitation combined with quantitative PCR (ChIP–qPCR) were employed in the MITE excision lines and the wild types. According to the bisulfite sequencing data and the ChIP-qPCR results, the DNA methylation and H3K9 dimethylation level at the *Ghd2* locus showed no significant difference between the MITE excision lines and wild types with the MITE (Supplementary Fig. 11). Meanwhile, our data suggested that the *Ghd2* transcript level is not affected by the presence or absence of MITE (Figs 1c and 2c), indicating that the MITE excision may not affect the transcription level of this gene.

It should be noted that 21–22-nt small RNAs were observed in the siRNA-overexpression lines and in the DCL3a RNAi lines (Fig. 3d and Supplementary Fig. 5a and b). It is possible that other Dicer-like proteins are involved to generate distinct sizes of small RNAs, likely through their coordinated actions with OsDCL3a (ref. 31). Previous data showed both 24-nt miRNAs and 21-nt miRNAs, derived from the same miRNA precursor, would direct DNA methylation and RNA cleavage, respectively^31^. These 21–22-nt small RNAs may be possible clues to further unveiling the MITE-mediated translational repression.

The sMITE-mediated *Ghd2* translational repression provides a novel mechanism to prevent the excessive accumulation of the Ghd2 protein in addition to the transcriptional regulation. As a gene controlling multiple agronomic traits, *Ghd2* is conserved in rice subspecies including *indica* and *japonica*. Additionally, we analysed the genomic sequence of *Ghd2* in wild rice, and found that the MITE in the *Ghd2* 3′ UTR is conserved in the wild relatives of the Asian cultivated rice, *O. rufipogon*, but not in the wild rice *O. meridionalis* (Supplementary Fig. 12).

Taken together, our findings reveal that MITE-mediated translational repression is an unreported but important mechanism for fine tuning gene expression at the translational level (Supplementary Fig. 10). Gene-specific regulation by MITEs, especially for agronomically important genes such as *Ghd2*, may represent an outcome of evolutionary pressure on the genomic organization of transposable elements. The MITE embedded in the *Ghd2* 3′-UTR, which regulates the protein level of Ghd2, may represent a promising engineering tool for controlling the protein levels of other agronomically important genes.

## Methods

### Plant materials

*Oryza sativa* L. ssp. japonica var. Zhonghua11 was used to generate *Ghd2* overexpression, *Ghd2* CRISPR, and MITE excision transgenic lines using *Agrobacterium*-mediated transformation^55^. Rice calli were induced from matured embryo. After subculture, the calli were co-cultured with *Agrobacte*rium strain EHA105 containing the constructs described below. The transformed calli were selected on the basis of hygromycin B (Roche) or G-418 (Promega) resistance to generate transgenic plants. In all of the transgenic lines, the copy numbers of marker genes for transformation were determined by Southern blot. Two to three independent single-copy lines and the segregated non-transgenic lines were used for phenotypic analysis in the T_2_ or T_3_ generation (Supplementary Table 1). To generate MITE excision lines with background close to the wild type, three independent T_2_ transgenic MITE excision plants were backcrossed with the wild type rice Zhonghua11. The backcrossed MITE excision plants (McF2) and non-excision plants (WT′) segregated from BC_1_F_2_ generation were identified for further experiments.

The *OsDCL1*, *OsDCL3a*, *OsDCL3b*, *OsRDR2* and *OsAGO4ab* RNAi lines and the *osdcl4* mutant were obtained from the authors of previous reports^31^,^56^,^57^. The wild rice materials were from Prof. Qifa Zhang's lab and the cultivated rice DNA was provided by the authors of a previous report^58^. The *OsDCL3a* RNAi construct^31^ was introduced into the *Ghd2* FL overexpression lines to obtain the *OsDCL3a*-RNAi *Ghd2* FL overexpression lines.

### Phenotypic analysis

All of the transgenic lines were grown during natural rice growing seasons in the experimental fields of Huazhong Agricultural University (114.36°E, 30.48°N), Wuhan, China. For the diurnal expression pattern analysis, 15-day-old seedlings of the overexpression lines and the WT lines were cultivated in a phytotron (PGV36, Conviron) at 60% humidity under long-day conditions (cycles of 14 h of light at 30 °C and 10 h of dark at 26 °C) or under short-day conditions (cycles of 10 h of light at 30 °C and 14 h of dark at 26 °C).

The flowering time (heading date) was the days from the germination to the spikelet head-out. The plant height was measured by the height from the ground surface to the top of the plant at the mature stage. The main panicle length and main panicle spikelet number was determined after collection. All of the phenotypic traits were measured for at least ten plants of each genotype with two replicates. The Student's *t*-test was used for statistical analysis.

### Protoplast isolation

For transient expression in rice protoplasts, 3-day-old seedlings of Zhonghua11 were planted on 1/2 MS medium and were cultivated in an illumination room at 60% humidity, with 14 h of light at 28 °C and 10 h of dark at 25 °C. Protoplast isolation was performed with 12 to 15-day-old seedlings^59^. The leaf sheaths of seedlings were digested with 1.5% Cellulase R-10 (Yacult Pharmaceutical) and 0.75% Macerozume R-10 (Yacult Pharmaceutical) for 5 h. After filtering through 40 μm nylon mesh, the protoplasts were collected and incubated in the W5 solution (154 mM NaCl, 5 mM KCl, 125 mM CaCl_2_, 22 mM MES, pH 5.7) at room temperature for at least 1 h. After incubation, the protoplasts were resuspended in the MMG solution (0.6 M mannitol, 15 mM MgCl_2_, 4 mM MES, pH 5.7), and the concentration of protoplasts was adjusted to the 1 × 10^7^ ml^−1^ for PEG-CaCl_2_ mediated transfection.

### Genotypic analysis

The genotypes of *Ghd2* and siRNA overexpression lines were identified by genomic PCR using the Hn forward and reverse primers (Supplementary Table 2). The genotypes of *OsDCL1*, OsDCL3a, *OsDCL3b*, *OsRDR2*, *OsAGO4ab* RNAi lines and *osdcl4* mutants were identified as per the methods utilized in previous reports^31^,^56^,^57^. The genotypes of MITE excision lines, wild rice, and rice cultivars were identified by genomic PCR using the MEC forward and reverse primers and the amplified genomic DNA was confirmed by sequencing (Fig. 2b and Supplementary Fig. 4b). The genotypes of *Ghd2* CRISPR lines were identified by sequencing using the Ghd2 crispr forward and reverse primer (Supplementary Fig. 1a). The foreigner DNA in the MITE excision lines was identified by PCR using the Cas9 forward and reverse primers (Supplementary Table 2).

### Plasmid construction

A full-length DNA fragment of *Ghd2* was amplified from Zhonghua11 genomic DNA with the Ghd2 fl forward and reverse primers. The full-length *Ghd2* without the 3′-UTR was amplified from the *Ghd2* full-length fragment with the *Ghd2* cds forward and reverse primers. The full-length *Ghd2* without the MITE was generated via fusion PCR using the Ghd2 fl primers and Ghd2 fldM inside primers. All of the amplified fragments were cloned into pCAMBIA1301H, in which the *Ghd2* genes were driven by the *OsLEA3* promoter^60^. To generate the *Ghd2* CRISPR lines, fragments containing single guide RNA (sgRNA) were amplified with the Ghd2 crispr primers and were cloned into pH-Ubi-cas9-7 (ref. 61). The sgRNA was designed to target the Ghd2 coding region (Supplementary Fig. 1a).

For the multi-sgRNA-directed transposable element excision, four sgRNAs flanking the sMITE were designed according to the CRISPR-PLANT database (www.genome.arizona.edu/crispr/)^62^. The locations and sequences of guide RNAs used for the CRIPSR construct were shown in Supplementary Fig. 4a and b. The transfer RNA and guide RNA scaffold were amplified from the pGTR vector with the Ghd2_TED primers (Supplementary Table 2), assembled using golden gate assembly strategy, and ligated into to the pRGEB32 vector^63^ for the *Agrobacterium*-mediated rice transformation.

For siRNAs overexpression, the OsmiR440 precursor was amplified from the rice genome with the OsmiR440 forward and reverse primers. The mature OsmiR440 sequence was replaced by siR381 and siR382 to construct artificial siRNA precursors with the siR381 and siR382 primers (Supplementary Table 2)^64^,^65^. The pCAMBIA1301U was employed to carry the artificial siRNA precusors, driven by the maize ubiquitin promoter^66^.

The reporter in p2CGW7 was replaced with the firefly or *Renilla* luciferase to generate p2FLGW7 or p2RLGW7 for the 3′-UTR assay with the Fluc fl and Rluc fl primers. The *Ghd2* 3′-UTR was obtained by PCR with the GU fl forward and reverse primers. The *Ghd2* 3′ UTR deletion mutants were generated from the full-length *Ghd2* 3′-UTR with the primers listed in Supplementary Table 2. The *Ghd2* 3′-UTR ΔM was generated with a strategy similar to that used for the *Ghd2* FLΔM. The 35S terminator, a negative control, was amplified from pJAM1849 with the 35S Ter forward and reverse primers. The wild-type sMITE (WM) was obtained by PCR with the WM forward and reverse primers. The sMITE mutants (MM.1, MM.2 and MM.3) were generated by primer-directed mutagenesis using the MM.1 forward primer, MM.2 reverse primer, and WM primers.

To replace the sMITE with other DNA fragments, a fragment from the 3′-UTR was amplified with the GUS.1 forward and reverse primers, and a Ty3/gypsy retrotransposon was amplified with the GUS.2 forward and reverse primers from rice genomic DNA. The *Ghd2* 3′ UTRΔM/pDONR221 fragment was amplified with the GUdM/p221 forward and reverse primers. The sMITE was substituted through blunt-end ligation between the *Ghd2* 3′-UTRΔM/pDONR221 and the replacing fragments. The three randomly chosen gene 3′-UTRs were amplified with the OU1-3 forward and reverse primers from rice genomic DNA.

The fragments used for transient expression were cloned into pDONR221 using the Gateway BP clonase II enzyme (Invitrogen) and were subsequently moved into p2FLGW7 with the Gateway LR clonase II enzyme (Invitrogen). The 35S Ter was cloned into p2RLGW7 for normalization.

A complete list of oligonucleotides used for plasmid preparation is in the Supplementary Table 2.

### RNA ligase-mediated rapid amplification of cDNA ends

The 3′ UTR of *Ghd2* was amplified from the reverse-transcribed cDNA of rice tissue using RNA ligase-mediated RACE (GeneRacer Kit, Invitrogen). The 3′ UTR was then cloned into a pGEM-T easy vector (Promega) and sequenced. The cleavage site was mapped using modified 5′ RNA ligase-mediated RACE with the GeneRacer Kit following the manufacturer's instructions. The ligation of an RNA oligomer to the mRNA 5′-end was followed by reverse transcription, amplification, cloning and sequencing. The primer sequences are listed in the Supplementary Table 2.

### Quantitative RT–PCR

Total RNA was isolated from rice leaves using TRIzol Reagent (Invitrogen) in accordance with the manufacturer's instructions. A 3 μg of RNA sample was subjected to deoxyribonuclease I treatment (Invitrogen) and reverse transcribed using SuperScript III Reverse Transcriptase (Invitrogen) with Oligo(dT)_18_ or gene specific reverse primers. Quantitative RT–PCR was performed in a StepOnePlus Real-Time PCR System (Applied Biosystems) using FastStart Universal SYBR Green Master (Roche). Ubiquitin was used as a reference gene in the qRT–PCR experiments. The transcripts were quantified with the comparative Ct method, and differences in gene expression were presented as the normalized fold expression (ΔΔCt). The qRT–PCR was performed three times independently. The primers used for the qRT–PCR are listed in the Supplementary Table 2.

### Western blotting

Rice young leaf samples were homogenized in liquid nitrogen and lysed in 500 μl of lysis buffer per 25 mg. Total proteins were separated in a 12% SDS polyacrylamide gel and were transferred to PVDF membranes (GE Healthcare). Blotting was performed with 1:2,000 anti-Ghd2 rabbit polyclonal antibody (NewEast Bioscience) and 1:1,000 anti-FLAG antibody (A2220, Sigma). Horseradish peroxidase (HRP) signals were detected after incubation with the recommended HRP-conjugated secondary antibodies by employing chemiluminescence (SuperSignal West Pico Chemiluminescent substrate, Thermo). The signal intensity was quantified with ImageJ (National Institutes of Health, http://rsb.info.nih.gov/ij/). The relative abundance was calculated from the quantified Ghd2 blotting signal intensity versus the quantified Rubisco signal intensity. All the western blotting was performed three times independently. Assessment of Ghd2 antibody specificity is shown in Supplementary Fig. 13. The original western blotting and gel images are provided in Supplementary Fig. 14.

### Dual-luciferase reporter assay in protoplasts

The p2FLGW7-3′ UTR and p2RLGW7 were co-transfected into rice protoplasts by PEG-CaCl_2_, and the protoplasts were lysed to perform a luciferase activity assay with the Dual Luciferase Reporter Assay System (Promega). In addition, RNA was isolated 14 h after transfection with TRIzol Reagent (Invitrogen). The RNA samples were reverse transcribed and quantified using quantitative RT–PCR. Each assay was performed three times independently in protoplasts.

### Small RNA northern blotting

Total RNA was isolated from rice leaf tissues using TRIzol Reagent. Low-molecular-weight (LMW) RNA was precipitated from the supernatant after precipitation with sodium chloride and polyethylene glycol (PEG) 8,000 (ref. 67). Then, 30 μg of LMW RNA was separated on a 15% denaturing PAGE gel and was transferred to a nylon membrane (GE Healthcare). Locked nucleic acid-modified probes and oligonucleotide probes (Exiqon; listed in Supplementary Table 2) were used for small RNA detection^68^. The signal intensity was quantified with ImageJ (National Institutes of Health, http://rsb.info.nih.gov/ij/). The relative abundance was calculated from the quantified siRNA blotting signal intensity versus quantified loading control (U6 or miR168). The original small RNA northern blotting images were provided in Supplementary Fig. 14.

### Bisulfite sequencing analysis

To determine the DNA methylation levels in the MITE excision lines and the wild types, the genomic DNA was isolated from leaves at 60-day after germination using the DNeasy Plant Mini Kit (QIAGEN). Bisulfite treatment was performed with the EpiTect Bisulfite kit (Qiagen) following the manufacturer's instructions. The treated DNA was used for PCR amplification with primers at the *Ghd2* locus. The primers were designed by Methyl Primer Express software (Applied Biosystems). Primers for bisulfite sequencing were listed in the Supplementary Table 2. All PCR fragments were cloned to pGEM-T easy vector (Promega) and 20 clones for each fragment were sequenced. The sequencing data were analysed on the web-based kismeth bisulfite analysis sofeware^69^.

### Chromatin Immunoprecipitation assay

To investigate the histone modification at the *Ghd2* locus in the MITE excision lines and the segregated wild types, chromatin immunoprecipitation (ChIP) assays were carried out in the mature leaves as previously described^70^. The antibodies for anti-H3K9 dimethylation (Abcam A1220) and mouse IgG2 (abcam 18413) were employed in the ChIP followed by qPCR. For validation of the ChIP results of H3K9me2, a rice Ty1-copia retrotransposon (*Os02g30880*) and the actin gene (*Os11g06390*) were used as positive and negative controls, respectively. The primers for ChIP–qPCR were listed in the Supplementary Table 2.

### Statistical analysis

The two-tailed Student's *t*-test was used for statistical analysis whenever two groups were compared. Statistical significance was determined at *P*<0.05 (*) or *P*<0.01 (**).

### Data availability

The sequences of p2FLGW7 and p2RLGW7 have been submitted to Genbank under accession code (KY434118 and KY434119). The authors declare that all data supporting the findings of this study are available within the manuscript and its supplementary files or are available from the corresponding author on request.

## Additional information

**How to cite this article:** Shen, J. *et al*. Translational repression by a miniature inverted-repeat transposable element in the 3′ untranslated region. *Nat. Commun.* **8,** 14651 doi: 10.1038/ncomms14651 (2017).

**Publisher's note:** Springer Nature remains neutral with regard to jurisdictional claims in published maps and institutional affiliations.

## Supplementary Material

Supplementary Information Supplementary Figures and Supplementary Tables
