# Supplementary material for: Translational repression by a miniature inverted-repeat transposable element in the 3′ untranslated region
Source: Nat Commun. 2017 Mar 3;8:14651. doi: 10.1038/ncomms14651 (PMC5338036; doi:10.1038/ncomms14651)
Supplement: Supplementary Information — Supplementary Figures and Supplementary Tables [file ncomms14651-s1.pdf]

Supplementary Figures

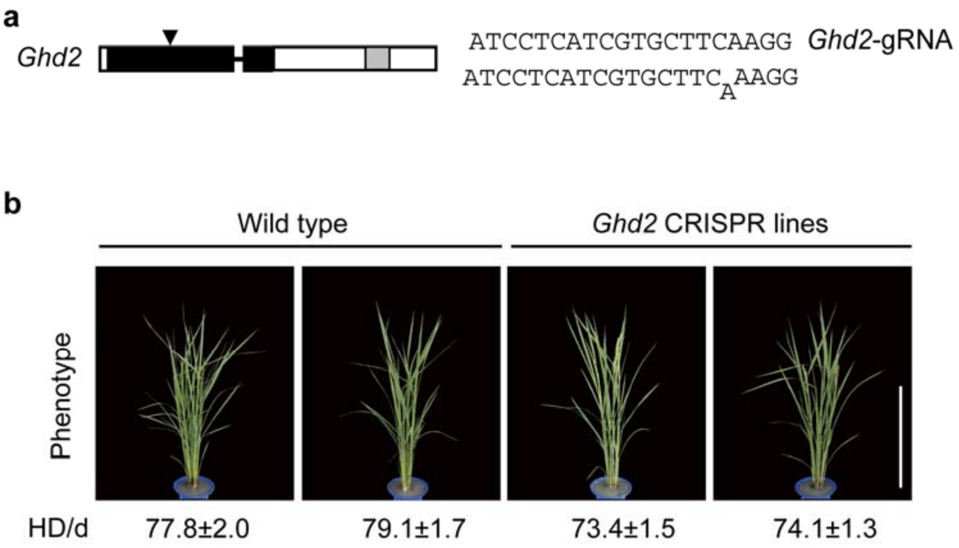

Supplementary Figure 1. Phenotypes of WT and *Ghd2* CRISPR lines at the heading stage. a, The sgRNA was used for *Ghd2* editing in the coding region. The black triangle indicates the sgRNA location. Exons are in black; 3′ and 5′ UTRs are in white; the transposable element (sMITE) is in gray; and the intron is indicated as a line. The CRISPR lines were confirmed by sequencing, shown in the right panel. **b,** Two independent transgenic lines with reading-frame-shifted mutation in the target region showed an earlier flowering time. Wild type is the segregated non-transgenic genotype. HD: heading date (days). Scale bar, 50 cm. The data represent the means ± s.d., n=10.

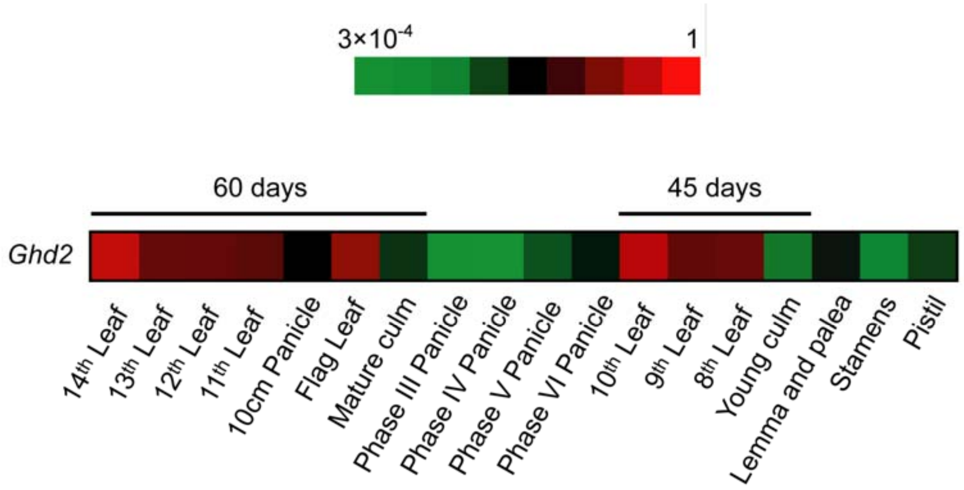

Supplementary Figure 2. Quantitative expression level analysis of *Ghd2* in rice tissues. Relative expression level is quantified based on $\Delta\Delta Ct/\Delta\Delta Ct_{max.}$ The 8th to 14th leaves were harvested according to phyllotaxy of rice. Phase III panicle, 1-2 mm; Phase IV panicle, 4-5 mm; Phase V panicle, 25 mm; Phase VI panicle, 60 mm. The numbers above the heatmap indicate the sample harvest day after germination.

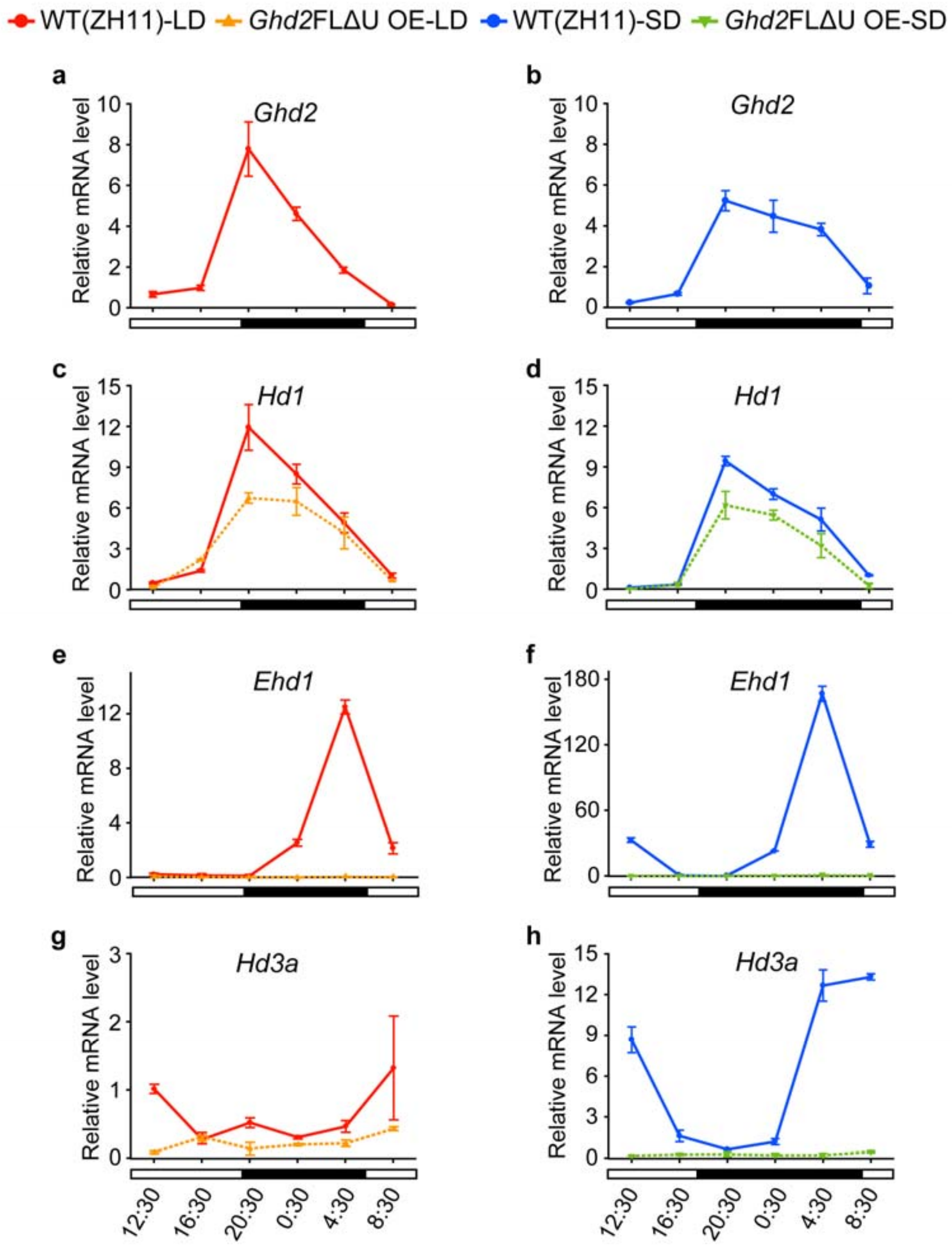

Supplementary Figure 3. Diurnal expression patterns of *Ghd2* and flowering genes in the wild type and *Ghd2* FLΔU lines examined by quantitative RT-PCR. **a,** *Ghd2* expression level under long day (LD) conditions. **b,** *Ghd2* expression level under short day (SD) conditions. **c,** *Hd1* expression level under LD conditions. **d,** *Hd1* expression level under SD conditions. **e,** *Ehd1* expression level under LD conditions. **f,** *Ehd1* expression level under SD conditions. **g,** *Hd3a* expression level under LD conditions. **h,** *Hd3a* expression level under SD conditions. Data indicate mean ± s.d., n=3. The black and white bars below each plot indicate the dark and light period of a day, respectively. The numbers under the bars in the plot **g** and **h** indicate the sample harvest time.

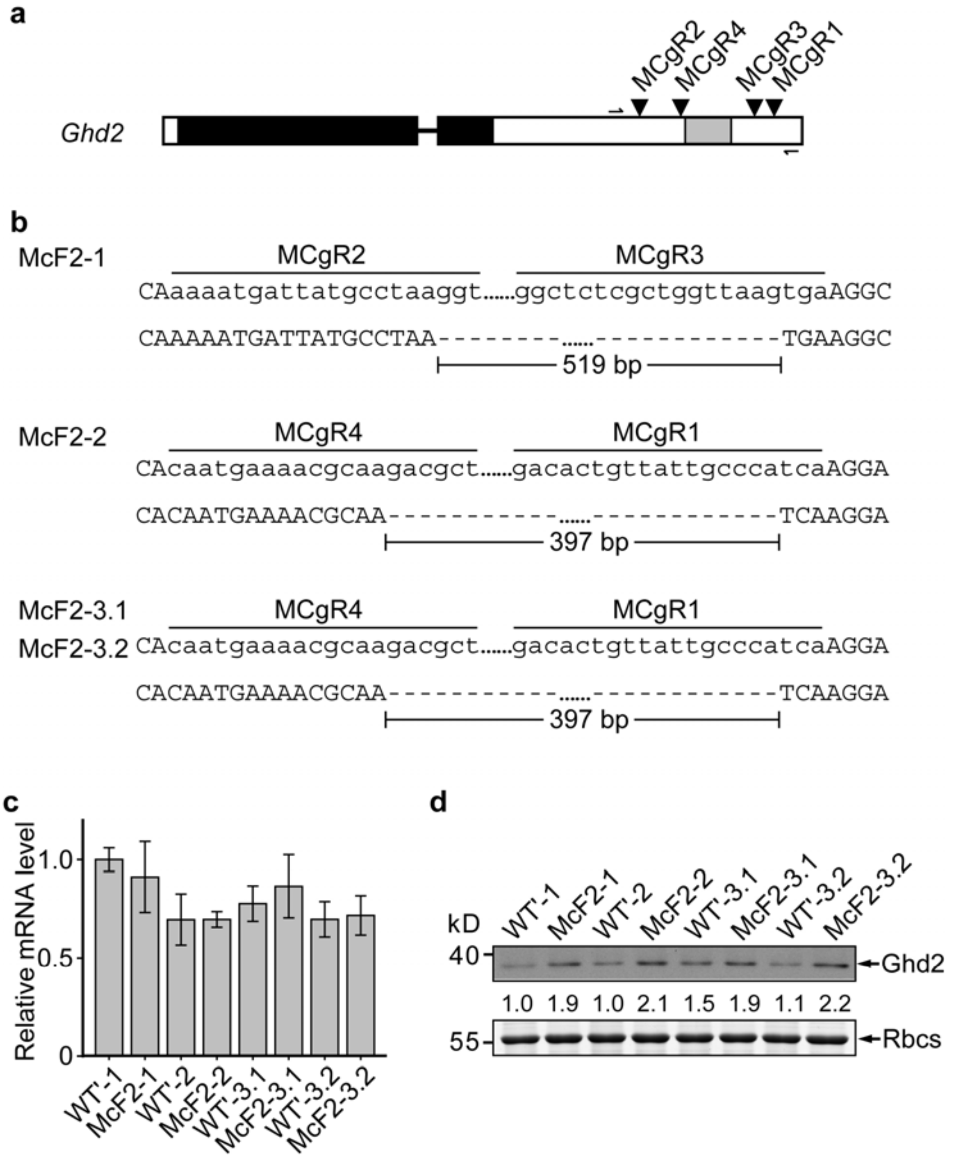

Supplementary Figure 4. The sgRNAs used for the sMITE excision and sMITE excision line genotyping and phenotyping. a, The location of sgRNAs in the *Ghd2* 3′ UTR. Exons are in black; 3′ and 5′ UTRs are in white; the transposable element (sMITE) is in gray; and the intron is indicated as a line. The black triangles indicate sgRNAs, and the arrows indicate the PCR primers for amplification. **b,** The excision region mediated by sgRNAs was confirmed by sequencing in the MITE excision lines. McF2-1, McF2-2, and McF2-3 are the names of excision lines segregated from the progenies (BC_1F_2) of the backcrosses between three different T_2 transgenic excision plants and wild type. **c,** Quantitative expression of *Ghd2* mRNA in the sMITE excision lines (McF2) and the corresponding WT′ segregated from the backcrosses. Another replicate of the experiment in Fig. 2c. The data represent the means ± s.d., n=3. **d,** The Ghd2 protein was measured by western blotting with the anti-Ghd2 antibody. Another replicate of the experiment in Fig. 2d. Rubisco (Rbcs) was loaded as a control. The numbers between two blots indicate relative abundance normalized by Rbcs for single samples.

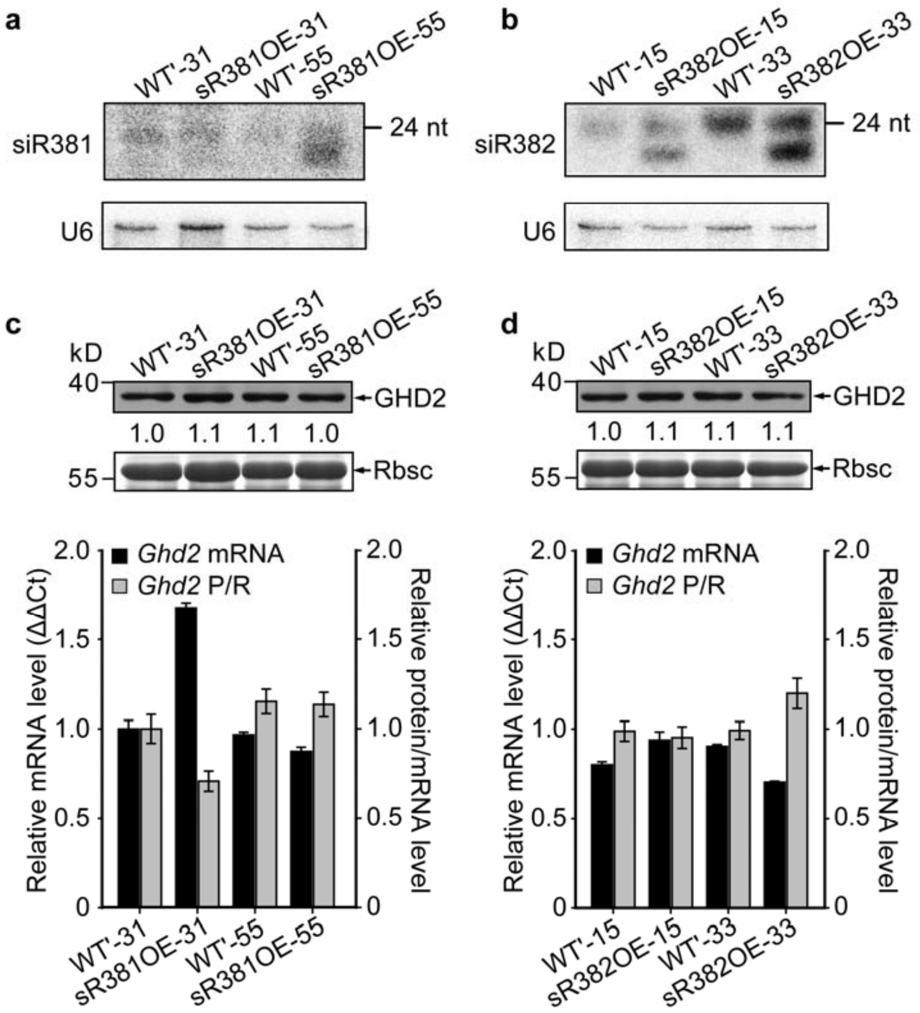

Supplementary Figure 5. The *Ghd2* mRNA and protein abundance in siRNAs over-expression lines. a and **b,** Small RNA northern blots to analyze the siRNA abundance in the siR381 and siR382 over-expression lines. **c** and **d**, *Ghd2* protein (upper panel) and mRNA abundance (lower panel) are shown. The WT′ indicates segregated non-transgenic line. The Ghd2 protein level was measured by Western blotting with the anti-Ghd2 antibody. Rubisco was loaded as a control. The numbers between two blots indicate relative abundance for single samples. The *Ghd2* relative expression level is based on $\Delta\Delta Ct/\Delta\Delta Ct_{max.}$ Relative protein/mRNA level means quantified protein abundance versus relative mRNA level. Data in **c** and **d** indicate mean ± s.d., n=3.

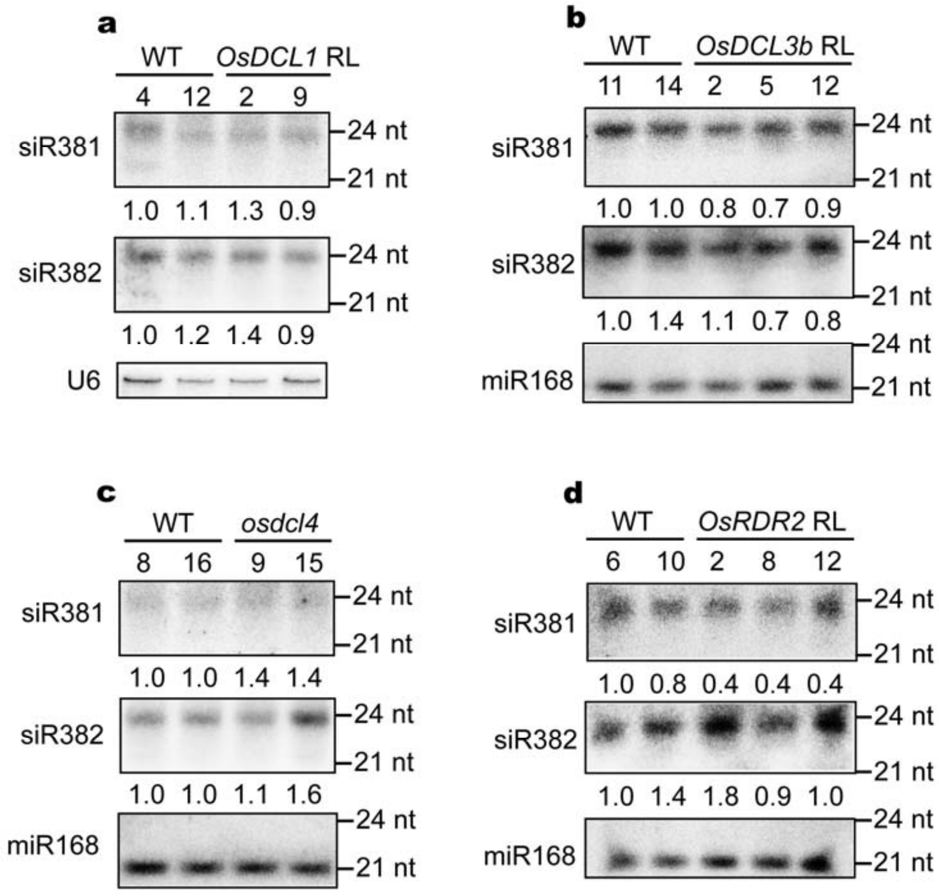

Supplementary Figure 6. Small RNA northern blots to determine siRNAs in the RNAi lines or mutants of rice *Dicer-like* (*OsDCL*) and *RNA-dependent RNA polymerase2* (*OsRDR2*) genes. a, *OsDCL1* RNAi lines (RL); **b,** *OsDCL3b* RNAi lines; **c,** *osdcl4* mutant lines; **d,** *OsRDR2* RNAi lines. In plot **a,** the U6 was probed as a loading control. In plots **b, c,** and **d,** the miR168 was probed as a loading control. The siR381 and siR382 probes are shown in the Figure 3a. The numbers under each siRNA blot indicate relative abundance.

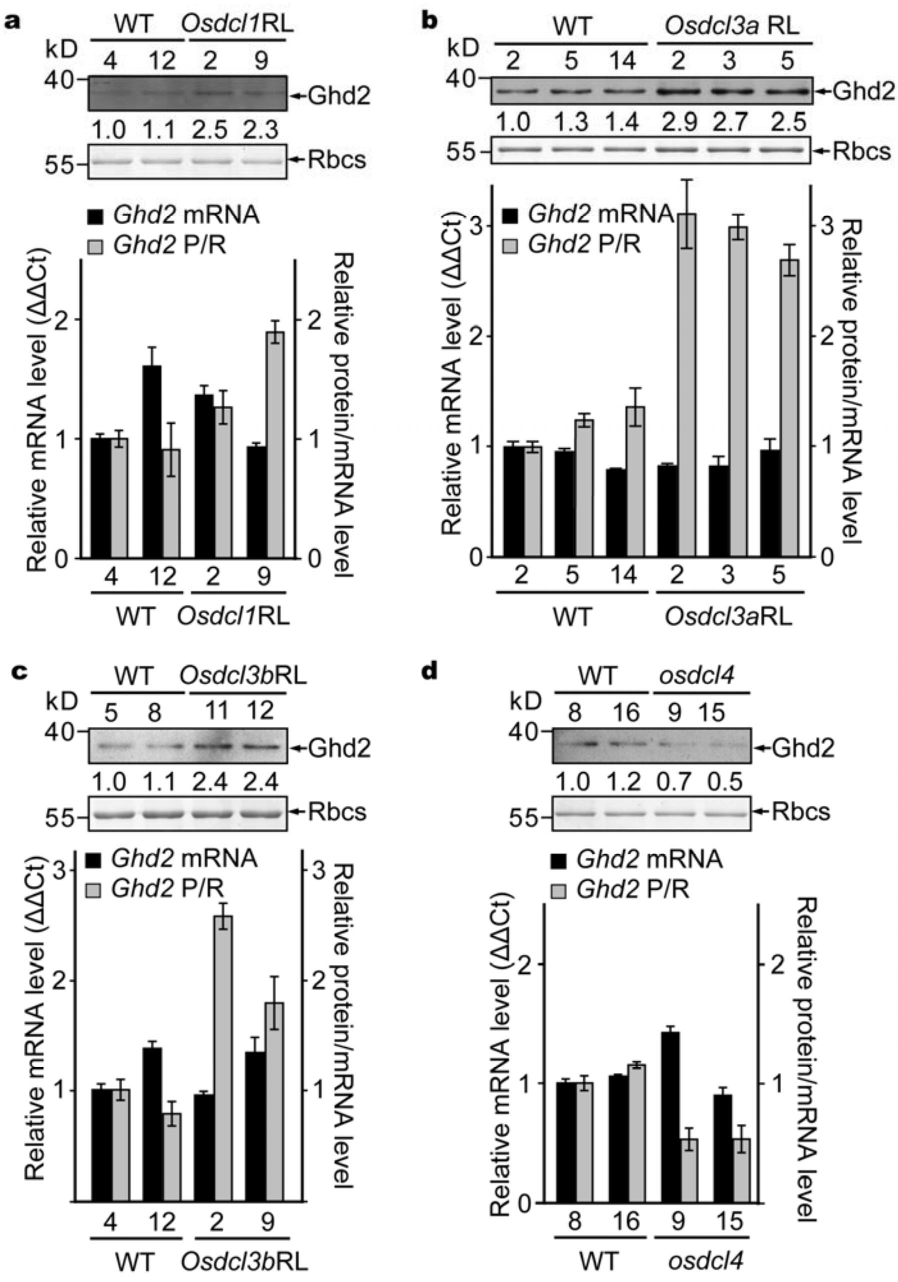

Supplementary Figure 7. Endogenous *Ghd2* mRNA and protein levels in the RNAi line or mutant of rice Dicer-like (OsDCL) genes. Western blots for *Ghd2* protein (upper panel) and qRT-PCR for *Ghd2* mRNA (lower panel) in *OsDCL1* **(a)**, *OsDCL3a* **(b)**, *OsDCL3b* **(c)** RNAi lines, and *osdcl4* **(d)** mutants. The Ghd2 protein level was measured by Western blotting with the anti-Ghd2 antibody. Rubisco was as a control. The numbers between two blots indicate relative abundance. The relative expression level is based on $\Delta\Delta Ct/\Delta\Delta Ct_{max.}$ Relative protein/mRNA level means quantified protein abundance versus relative mRNA level. Data indicate mean ± s.d., n=3.

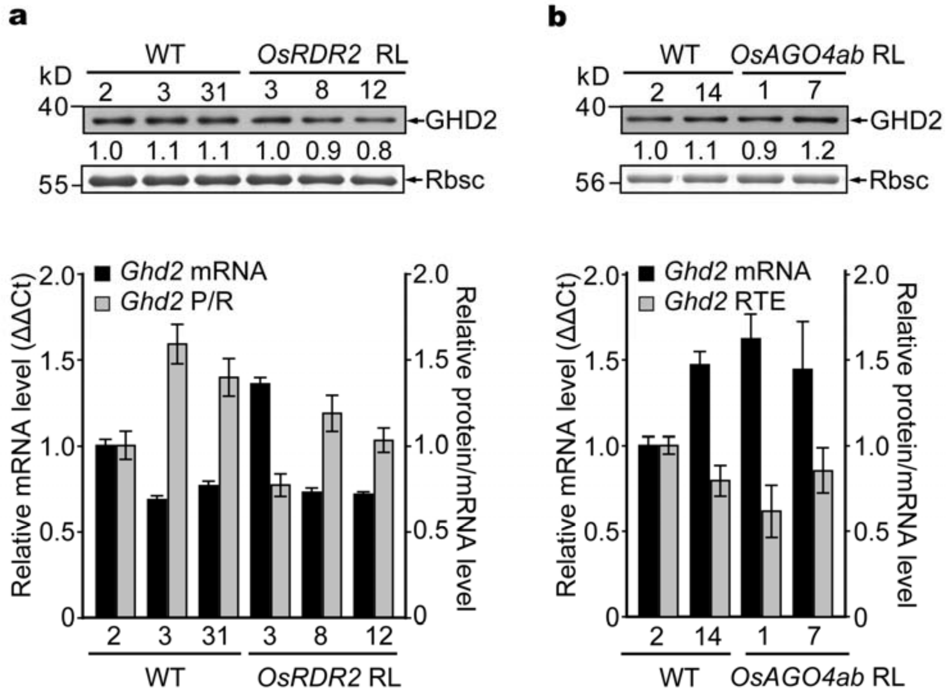

Supplementary Figure 8. Endogenous *Ghd2* mRNA and protein levels in *OsRDR2* and *OsAGO4ab* RNAi lines. Western blotting for *Ghd2* protein (upper panel) and qRT-PCR for *Ghd2* mRNA (lower panel) in *OsRDR2* **(a)** and *OsAGO4ab* **(b)** RNAi lines. The Ghd2 protein level was measured by Western blotting with the anti-Ghd2 antibody. Rubisco was as a loading control. The numbers between two blots indicate relative abundance for single samples. The relative expression level is based on $\Delta\Delta Ct/\Delta\Delta Ct_{max}$. Relative protein/mRNA level means quantified protein abundance versus relative mRNA level. Data indicate mean ± s.d., n=3.

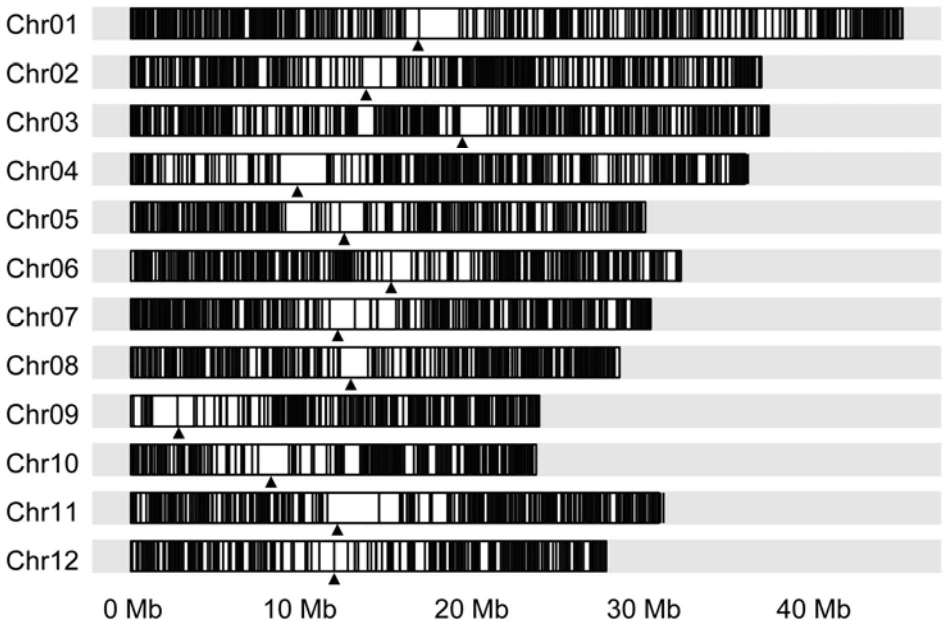

Supplementary Figure 9. A karyogram of OsT38 distribution in rice genome. Black line represents the OsT38. The triangles indicate centromeres.

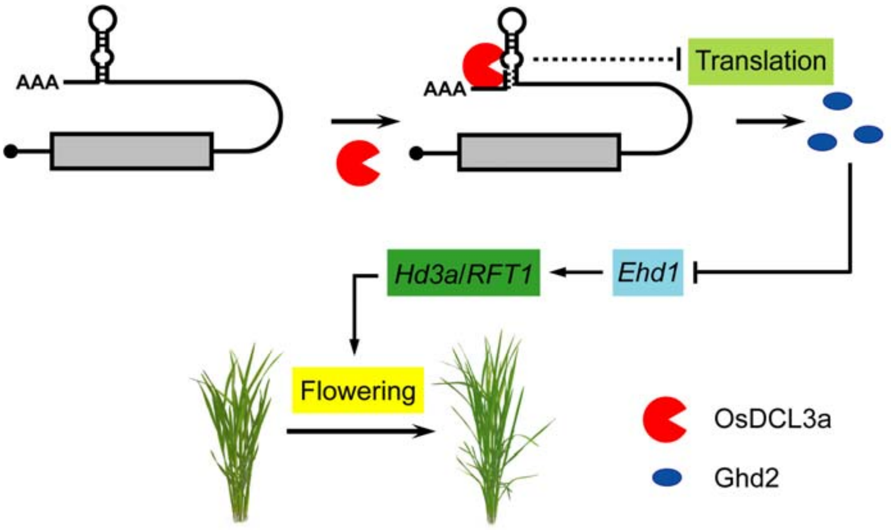

Supplementary Figure 10. A hypothetical model of MITE-mediated translational repression to suppress rice flowering.

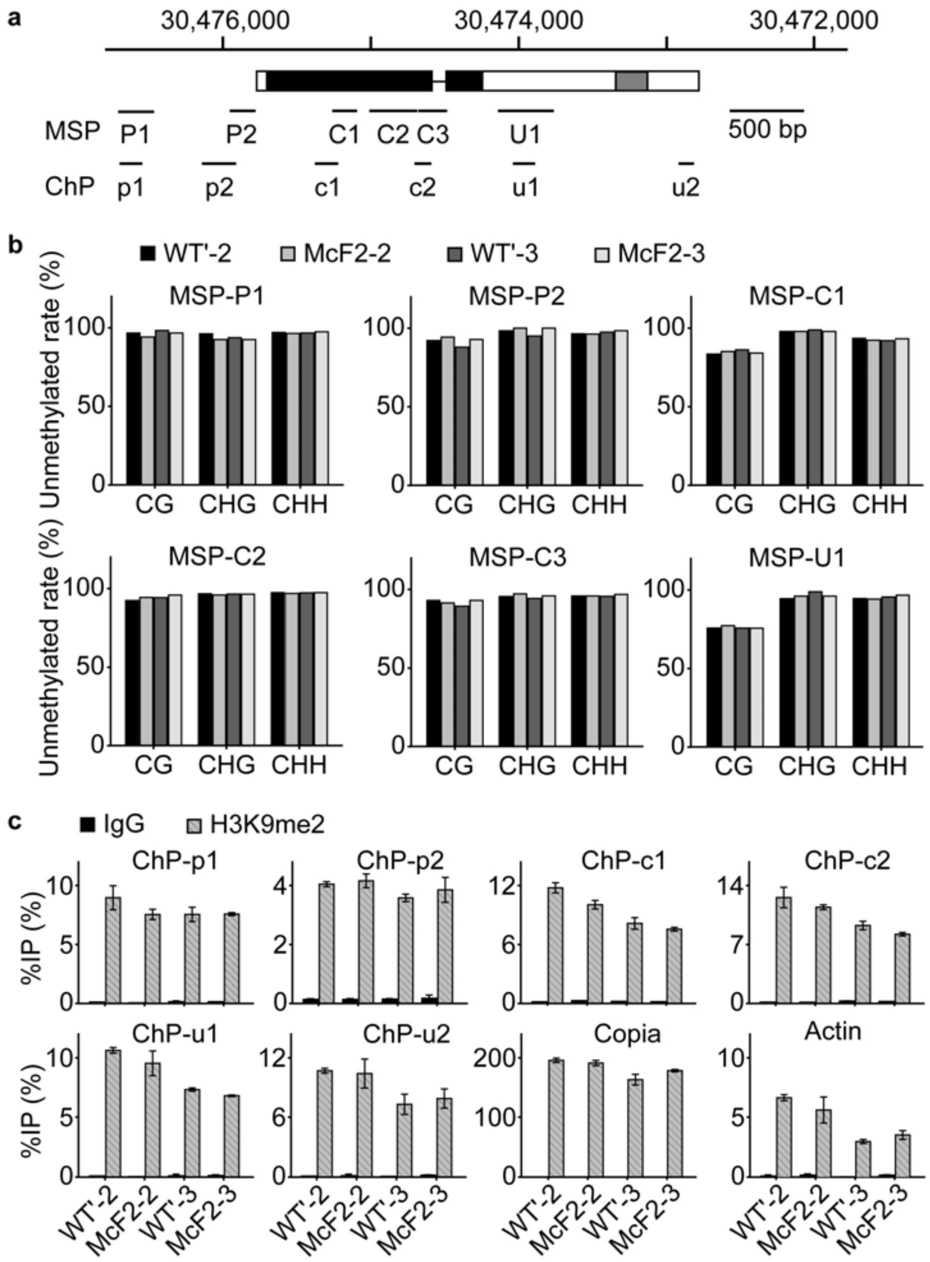

Supplementary Figure 11. The DNA methylation and H3K9 dimethylation levels in the *Ghd2* locus. **a,** Coding and untranslated regions of *Ghd2* gene are shown in black and white respectively; the transposable element (sMITE) is indicated in gray; and the intron is indicated as a line. The regions for bisulfite sequencing and the primers for ChIP-qPCR are indicated as MSP and ChP respectively. **b,** Bisulfite sequencing analysis of the DNA methylation level at the *Ghd2* locus. The CG, CHG, and CHH (H=A, T, or G) unmethylation levels are shown according to the sequencing data. WT', wild types without excision of the sMITE segregated from the CRISPR lines. **c,** The H3K9 dimethylation level at the Ghd2 locus. A rice Copia retrotransposon and the actin gene are used as the positive and negative controls, respectively, for the H3K9me2. The ChIP signals are indicated by gray bars. The signals from the mouse IgG control are indicated by black bars. The ChIP results are indicated as percentage of input (%IP). Data indicate mean ± s.d., n=3.

a

Name/Accession	MITE insertion	Country	Subpopulation
Safut Khosha	yes	Afghanistan	*Aus*
SORNAVARI	yes	Mali	*Aus*
Thang 10	yes	Vietnam	*Ind_Int*
Shui Ya Jien	yes	Hong Kong	*Ind_Int*
Mayang Khang	yes	Indonesia	*IndI*
YOU-I B	yes	China	*IndI*
BKN 6987-68-14	yes	Thailand	*IndII*
RP2151-173-1-8	yes	India	*IndII*
Manga Kely 694	yes	Madagascar	*Int*
A 152	yes	Bangladesh	*Int*
Vary Tarva Osla	yes	Portugal	*Jap_Int*
Pergonil 15	yes	Portugal	*TeJ*
Tamanishiki	yes	Japan	*TeJ*
NANTON NO. 131	yes	Taiwan, China	*TrJ*
YD1-2575	yes	China	*O. rufipogon*
IGRC105422	yes	Sri Lanka	*O. rufipogon*
IGRC105708	yes	India	*O. nivara*
IGRC105304	no	—	*O. meridionalis*

b

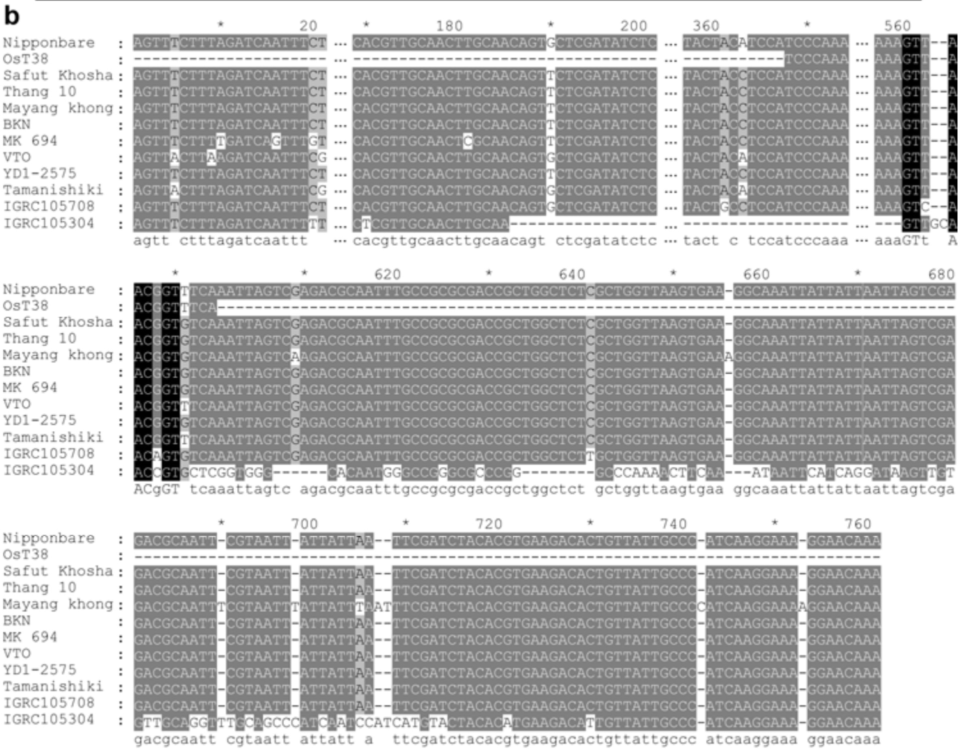

Supplementary Figure 12. The alignment of *Ghd2* 3' UTR sequences from cultivated and wild rice. a, The summary of the *Ghd2* 3' UTR genomic organization in cultivated and wild rice. **b,** The DNA sequences alignment of representative cultivated and wild rice. The OsT38 indicates the sMITE sequences.

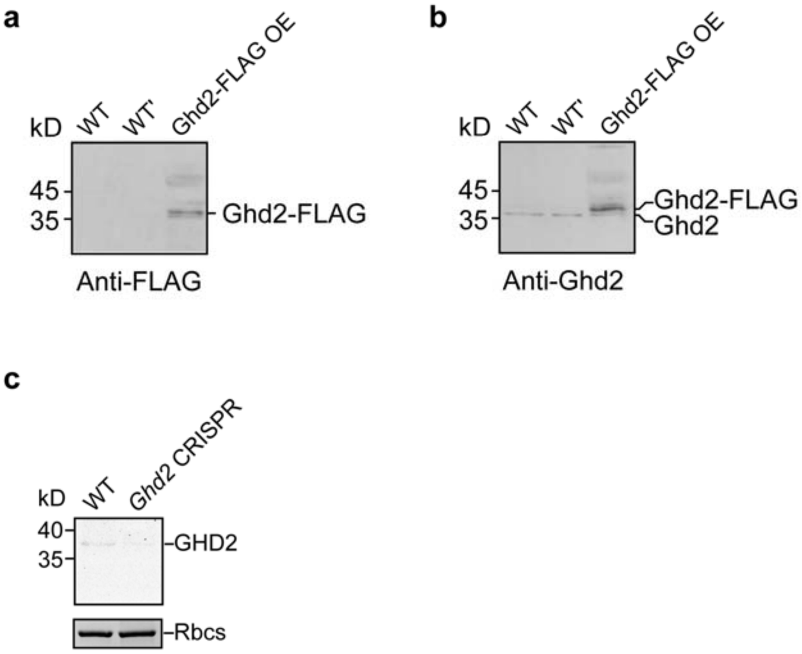

Supplementary Figure 13. Specificity of anti-Ghd2 antibody. The anti-Ghd2 antibody was validated in the Ghd2-FLAG transgenic line. **a,** anti-FLAG (Sigma), 1:3,000 dilution. **b,** anti-Ghd2, 1:1,000 dilution. **c,** anti-Ghd2, 1:2,000 dilution. Horseradish peroxidase (HRP) conjugated anti-mouse IgG and anti-rabbit IgG antibodies were employed as secondary antibodies.

Fig.1d

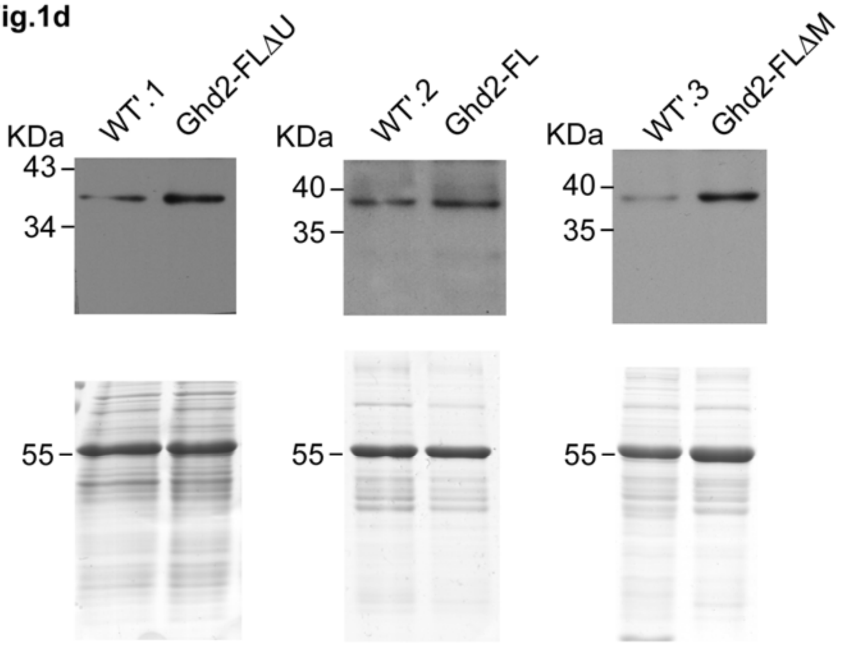

Fig.2b

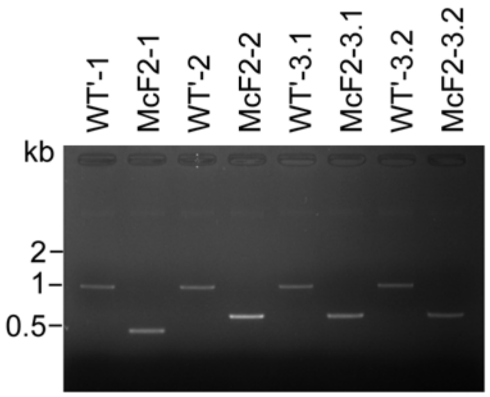

Fig.2d

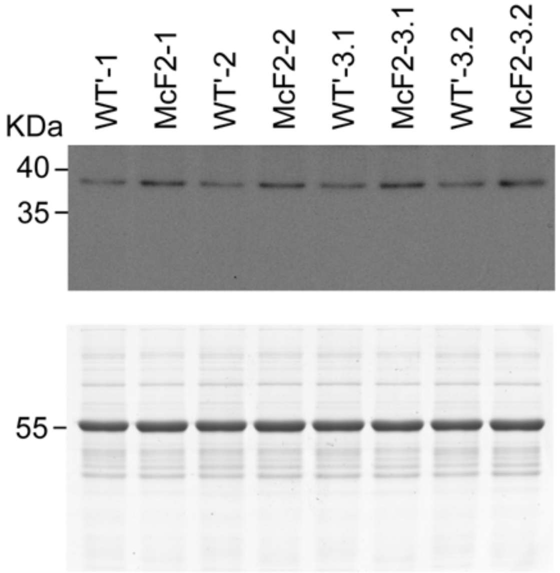

Fig. 3d

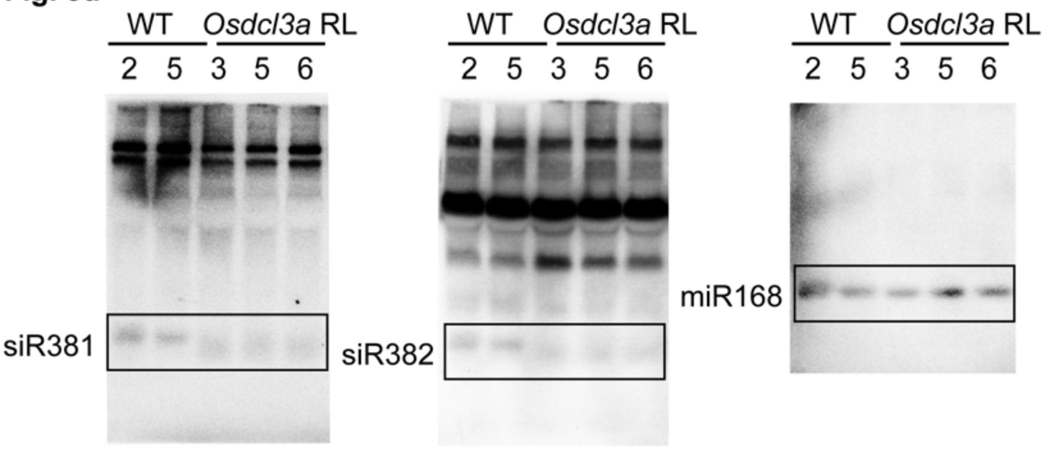

Fig. 3f

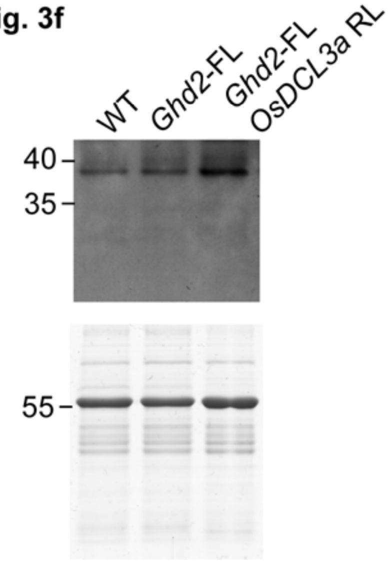

SFig. 4d

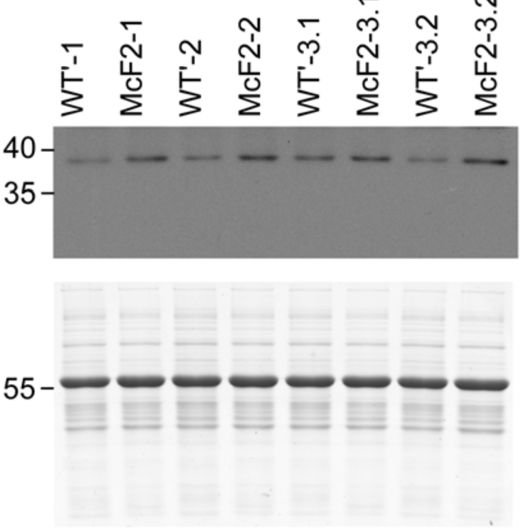

SFig. 5a

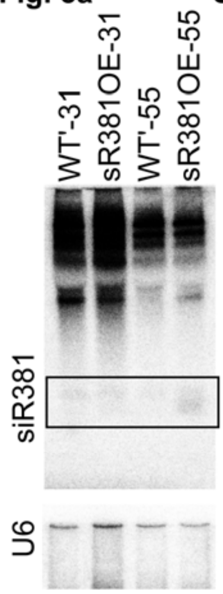

SFig. 5b

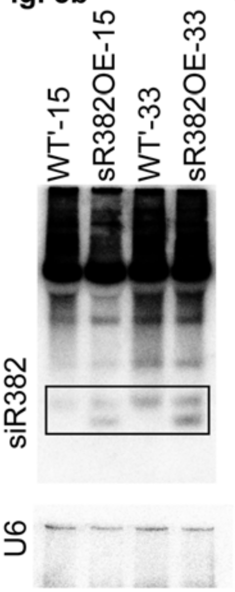

SFig. 5c-d

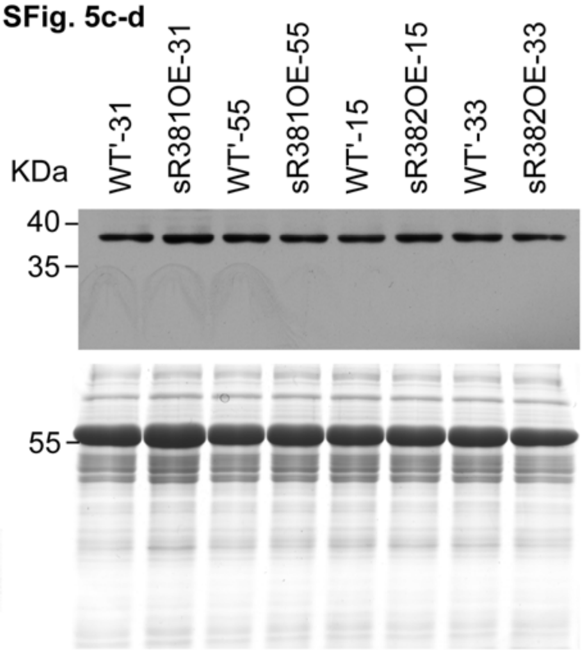

SFig. 6

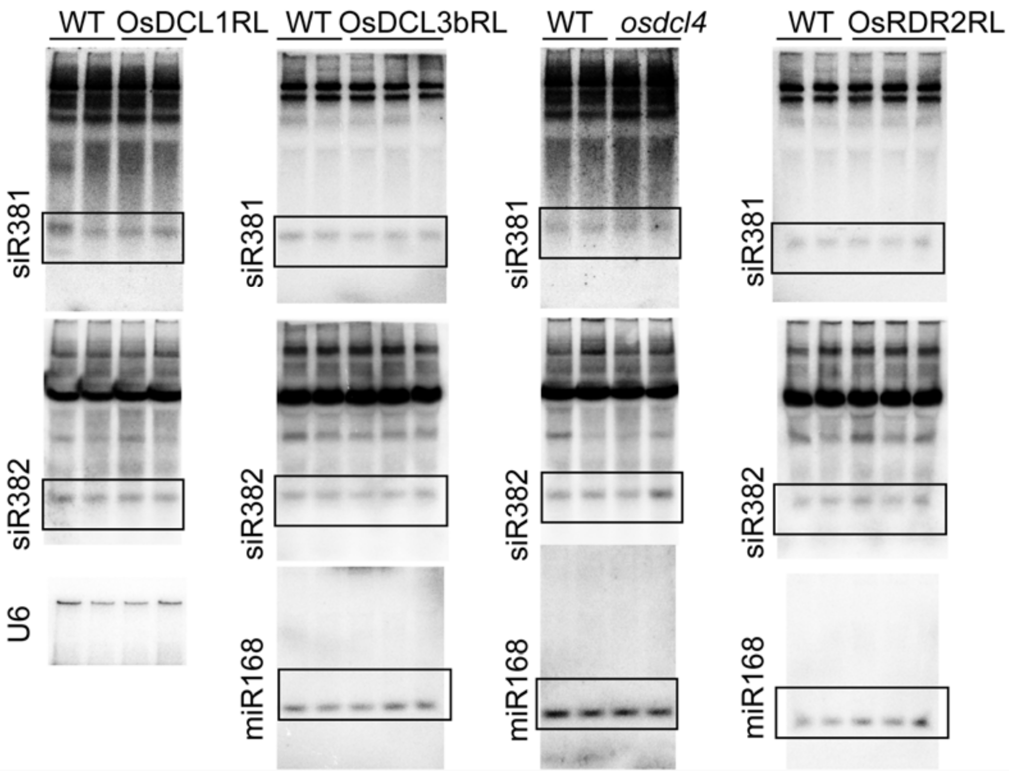

SFig. 7a

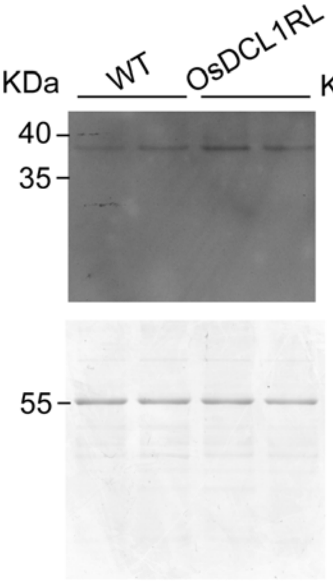

SFig. 7b

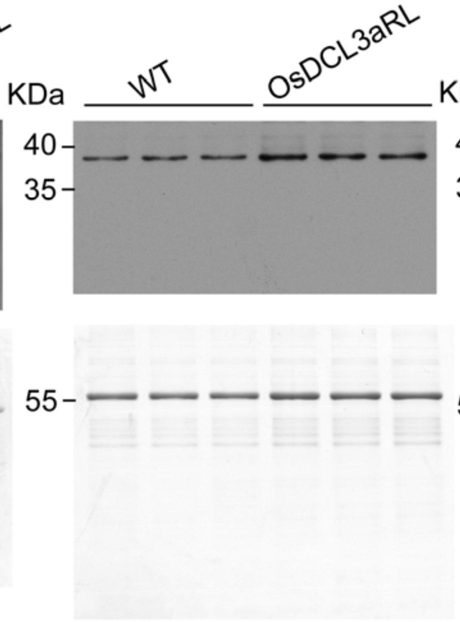

SFig. 7c

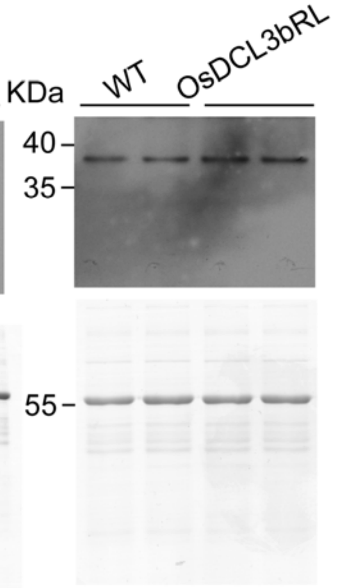

SFig. 7d

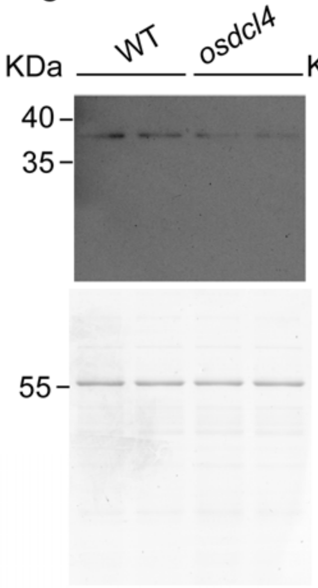

SFig. 8a

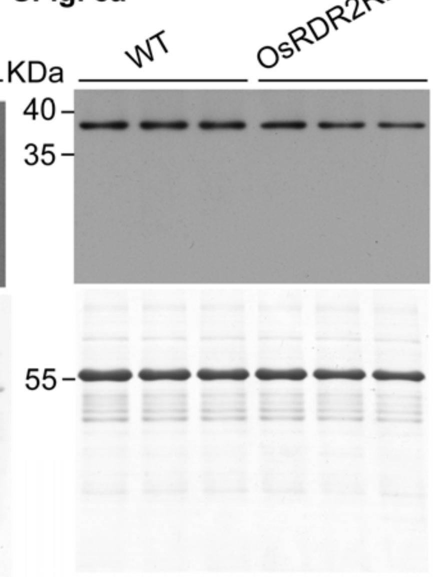

SFig. 8b

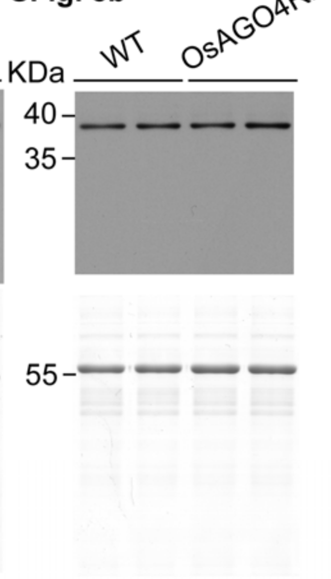

SFig. 13a

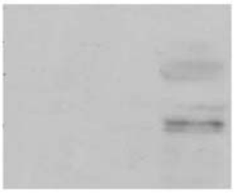

SFig. 13b

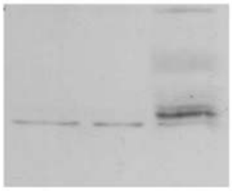

SFig. 13c

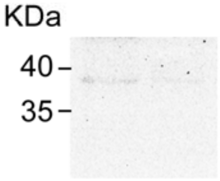

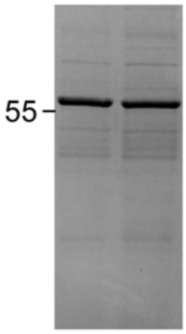

Supplementary Figure 14. The original western blots, northern blots, and gel images. The black frames indicate the portion showed in figures.

Supplementary Table 1. Flowering time, plant height, and grain number of main panicle of *Ghd2*-FLΔU, *Ghd2*-FL, and *Ghd2*-FLΔM and their segregated non-transgenic lines.

Genotype	Line#	Number of days to heading	Plant height (cm)	grain number of main panicle
Ghd2 FLΔU +	16	127.1±2.8	107.8±1.9	216.8±13.1
	21	119.7±3.0	111.5±2.9	224.6±7.6
	15	132.9±1.7	107.0±2.3	219.9±9.7
Ghd2 FLΔU -	16	78.8±1.2	85.2±1.7	170.1±7.2
	21	79.0±1.0	84.9±1.6	168.1±5.7
	25	79.4±0.9	84.5±1.5	173.9±11.4
Ghd2 FL +	40	93.8±1.1	105.8±3.2	179.1±6.9
	48	94.7±1.3	103.6±3.5	181.9±5.2
	58	97.9±1.6	102.0±2.7	180.5±7.5
Ghd2 FL -	40	79.7±0.7	84.5±1.3	186.0±6.1
	48	79.0±0.9	85.5±0.8	185.0±7.1
	58	79.3±0.6	84.9±1.7	189.4±7.3
Ghd2 FLΔM +	35	113.3±2.2	107.8±3.3	209.5±11.1
	43	109.7±1.6	105.6±2.7	210.2±14.7
	71	119.2±2.8	109.4±2.4	213.7±12.2
Ghd2 FLΔM -	35	79.4±0.9	85.2±2.1	182.4±9.5
	43	78.4±1.0	84.4±1.4	187.7±8.8
	71	79.8±1.1	85.8±2.0	184.5±9.2

Three independent transgenic lines were used for investigation in normal rice growing season at Wuhan, China. Data indicate mean ± s.d., n=20.

Supplementary Table 2. Primer lists

Primer name	Primer sequence
**Cloning primer**	**Sequence 5'->3'**
Ghd2 fl F BamHI	ggatccTCTTTTGTGCTGGGAGATTTCTTCTGTT
Ghd2 fl R XbaI	tctagaGAGGATTGCTGGGACAGAGTAAAAAATG
Ghd2 cds F KpnI	ggtaccCTGGGAGATTTCTTCTGTTT
Ghd2 cds R BamHI	ggatccTCGAGTCCATGAGGCATA
Ghd2 fldM F inside	GCGTCTCGACTAATTAATACTGGATGTAGTAATAATTTGCCGCGCGA
Ghd2 fldM R inside	GCAAATTATTACTACATCCAGTATTAATTAGTCGAGACGCAATTTGCCGC
Ghd2 crispr F	GGCAATCCTCATCGTGCTTCAAGG
Ghd2 crispr R	AAACCCTTGAAGCACGATGAGGAT
Ghd2_TED PS1 F	CGGGTCTCAcaataacagtgtctgcaccagccggg
Ghd2_TED PS1 R	TAGGTCTCCATTGCCCATCAgttttagagctagaa
Ghd2_TED PS2 F	CGGGTCTCAcataatcatttttgcaccagccggg
Ghd2_TED PS2 R	TAGGTCTCCTATGCCTAAGGTgttttagagctagaa
Ghd2_TED PS3 F	CGGGTCTCAccagcgagagcctgcaccagccggg
Ghd2_TED PS3 R	TAGGTCTCCCTGGTTAAGTGAgttttagagctagaa
Ghd2_TED PS4 F	CGGGTCTCAgcgttttcattgtgcaccagccggg
Ghd2_TED PS4 R	TAGGTCTCCACGCAAGACGCTgttttagagctagaa
OsmiR440 F	TTggtaccTCTGGACTTTCATGTACTCCTGCT
OsmiR440 R	TTggatccGGATAAGGGTCTGAAACAGGGAT
siR381 s	TTGAATAAGACGAACGGTCAAACATGGCTAACACCGATGAGA
siR381 as	CCATGTTTGACCGTTCGTCTTATTCAAATACATAAGTTTAG
siR381* s	GCATGTTTGACCGTTCGTCTTATTCAGATCAAGCTAGGCCT
siR381* as	TCTGAATAAGACGAACGGTCAAACATGCCCAGCACCAACAATA
siR382 s	TTAAAAAGTCAACGGCGTCAAACATTGGCTAACACCGATGAGA
asiR382 as	CCAATGTTTGACGCCGTTGACTTTTTAATACATAAGTTTAG
asiR382* s	GCAATGTTTGACGCCGTTGACTTTTTGATCAAGCTAGGCCT
asiR382* as	TCAAAAAGTCAACGGCGTCAAACATTGCCCAGCACCAACAATA
Fluc fl F	actagtATGGAAGACGCCAAAAACATAAAGAAAGG
Fluc fl R	ggtaccTTACACGGCGATCTTTCCGCCCT
Rluc fl F	actagtATGACTTCGAAAGTTTATGATCCAGAACA
Rluc fl R	ggtaccTTATTGTTCATTTTTGAGAACTCGCTCA
GU fl F	ggacaagtttgtacaaaaaagcaggctaaCACCATGCGTACGTGTCC
GU fl R	ggggaccactttgtacaagaaagctgggtaGAGGATTGCTGGGACAGAGTAA
GU d5.1 F	ggacaagtttgtacaaaaaagcaggctaaCCGGAGAGAAAGTAGGCGATT
GU d3 R	ggggaccactttgtacaagaaagctgggtaATAATAATTTGCCTTCACTTAACCAGC
GU d5.2 F	ggacaagtttgtacaaaaaagcaggctaaATGTAGTAGCTTGAATTTGCACGTTG
GUS.1 F	GGTGGCCATCTGAGCCCTGAGG
GUS.1 R	TTTACCAAAATGCCAGCAGGCGTAGT
GUS.2 F	TTAATTAGCAACATTAACTCCGATGCTCTGT

GUS.2 R	TGTCACAACCGGAGTTTTCCCCTTT
GUdM/p221 F	GTATTAATTAGTCGAGACGCAATTTGCC
GUdM/p221 R	TGGATGTAGTAATAATTTGCCGCGCGACA
35S Ter F	ggacaagtttgtacaaaaaagcaggctaaCGCTGAAATCACCAGTCTCTCT
35S Ter R	ggggaccactttgtacaagaaagctgggtaCGGTGTGAGGGAACTAGTTTTGAT
WM F	ggacaagtttgtacaaaaaagcaggctaaTCCCAAAATGTTTGACGCCGT
WM R	ggggaccactttgtacaagaaagctgggtaTCCCAAAATGTTTGAAACCGTTAAC
MM.1 F	ggacaagtttgtacaaaaaagcaggctaaTCCCAAAATGTTTGACGCCGTTGACTTTTTAACCAAT
MM.2 R	ggggaccactttgtacaagaaagctgggtaTCCCAAAATGTTTGAAACCGTTAACTTTTTTACCCAT
OU1 (Os03g60890) F	ggacaagtttgtacaaaaaagcaggctaaAAATCAATATCAGCCCGATTTACC
OU1 R	ggggaccactttgtacaagaaagctgggtaTTAACCCTCAAATAACCCAATCTGA
OU2 (Os01g71240) F	ggacaagtttgtacaaaaaagcaggctaaTTTTTGCAGGCATTTTGATCATCGT
OU2 R	ggggaccactttgtacaagaaagctgggtaTGAAGGACTATTGTGGAGTTAAAGTATACGATC
OU3 (Os01g43480) F	ggacaagtttgtacaaaaaagcaggctaaTGAAGAATTCATTACTTGTATATACATTTG
OU3 R	ggggaccactttgtacaagaaagctgggtaCATCCAGGGTTCGTCGGCGTC
**RACE primer**	**Sequence 5'->3'**
5' RLM-RACE R1	GAAGAGGATTGCTGGGACAGAGTAAAA
5' RLM-RACE R2	TTCCTTTCCTTGATGGGCAATAACAGT
3' RACE F1	CCGCTGGCTCTCGCTGGTTAAGTGAA
3' RACE F2	TGCCCATCAAGGAAAGGAACAAAACAAAG
**qRT-PCR primer**	**Sequence 5'->3'**
Ghd2 qPCR F	TTGCCTCCAACAGCAGGG
Ghd2 qPCR R	GCTTCGGATGAGCGCG
Ehd1 qPCR F	GGATGCAAGGAAATCATGGA
Ehd1 qPCR R	AATCCCATCGGAAATCTTGG
Hd1 qPCR F	TCAGCAACAGCATATCTTTCTCATCA
Hd1 qPCR R	TCTGGAATTTGGCATATCTATCACC
Hd3a qPCR F	CTTCAACACCAAGGACTTCGC
Hd3a qPCR R	TAGTGAGCATGCAGCAGATCG
Fluc qPCR F	TTTTGGAGCACGGAAAGACG
Fluc qPCR R	CTTCGTCCACAAACACAACTCC
Rluc qPCR F	ATCGGACCCAGGATTCTTTTCC
Rluc qPCR R	CATCAGGTGCATCTTCTTGCG
Ubi qPCR F	AACCAGCTGAGGCCCAAGA
Ubi qPCR R	ACGATTGATTTAACCAGTCCATGA
**Genotypic primer**	**Sequence 5'->3'**
Hn F	TACACAGCCATCGGTCCAGA
Hn R	TAGGAGGGCGTGGATATGTC
MEC F	GCTGTAAGCATTGTCCGCTAACAT
MEC R	GAGGATTGCTGGGACAGAGTAAAA

Ghd2 cripsr amp F	TTTGTGCTGGGAGATTTCTT
Ghd2 cripsr amp R	GCCTCGTAGTTGAGCCTGA
Ghd2 cripsr seq	TCGGTGGAGGAGGAGGCG
Cas F	CGGGATAAGCCCATCAGAGAGC
Cas R	GTAAAACGACGGCCAG
**bisulfite seq primer**	**Sequence 5'->3'**
MSP P1 F	AATAAGATAGGGATAGTATAATTGTAAGGA
MSP P1 R	ATCAATAAACTTTTAAAATAAAAACAAT
MSP P2 F	YGGAAAAGAGTATTTTATTTGGA
MSP P2 R	AAAAACCTCTAAAAATCTATACAACTCACT
MSP C1 F	AGTTYGAGGGGTAGTTGTTGTA
MSP C1 R	GCRGCAACTACRGAATACTTT
MSP C2 F	GGAAGTTTTATTYGGATAAGGT
MSP C2 R	AATTAAACCTAAAATCCAAACTCCTATAAA
MSP C3 F	GATTATYGAGAGTTGGGGTAAT
MSP C3 R	AACCCCTACACCAACACAATTT
MSP U1 F	TATGGATTYGATTGGTATAAAAG
MSP U1 R	TAATRGTCCRGATAAATTTTCAA
**ChIP-qPCR primer**	**Sequence 5'->3'**
ChP p1 F	GGGATAGCACAACTGCAAGG
ChP p1 R	TAGTGAGCCCCACCGATAAG
ChP p2 F	TTCCACCCACGTGTCATAAA
ChP p2 R	CGGATAAGGGCCATACTTGA
ChP c1 F	CAGGTCAAGAGCGTCGGGC
ChP c1 R	GCGAGGGAGGGGTCAAAGAC
ChP c2 F	CAGGCTCAACTACGAGGCGAT
ChP c2 R	CCGAGTAATGGTGGTCGTTGG
ChP u1 F	TCGGTGCTTTTGGATCTCTT
ChP u1 R	CAGGCACAACCCAGTTTACA
ChP u2 F	AACAACGGGTTTTGGTCTCTGC
ChP u2 R	TTGAAGAAGAGGATTGCTGGGAC
Copia F	TTTAGATGACATCCCATATGTTCTTTTC
Copia R	GGATGTTAAGACGGCATTCCTAAA
Actin F	TGCGTCAGGAATTCAGAACCA
Actin R	AGCACCACGAACCTTGACCAT
**Probe name**	**Sequence 5'->3'**
sR381 probe	T+GA+CC+GTT+CGT+CTTATT+CA
sR382 probe	AAT+GTT+TGA+CGC+CGTTGA+CTTT
miR168 probe	GTCCCGATCTGCACCAAGCGA
U6 probe	TGTATCGTTCCAATTTTATCGGATGT

In the locked nucleic acid (LNA) probe, the LNA-modified nucleotide is shown as +N.
